# Dynamic electrophoretic fingerprinting of the HIV-1 envelope glycoprotein

**DOI:** 10.1186/1742-4690-10-33

**Published:** 2013-03-20

**Authors:** Daniel J Stieh, Joshua L Phillips, Paul M Rogers, Deborah F King, Gianguido C Cianci, Simon A Jeffs, Sandrasegaram Gnanakaran, Robin J Shattock

**Affiliations:** 1 Center for Infection, Department of Cellular and Molecular Medicine, St George’s, University of London, London, SW17 0RE, UK; 2 Theoretical Division, Los Alamos National Laboratory, Los Alamos, New Mexico, USA; 3 Mucosal Infection & Immunity Group, Section of Infectious Diseases, Imperial College London, St Mary’s Campus, London, W2 1PG, UK; 4 Department of Cell and Molecular Biology, Feinberg School of Medicine, Northwestern University, Chicago, IL, 60611, USA

## Abstract

**Background:**

Interactions between the HIV-1 envelope glycoprotein (Env) and its primary receptor CD4 are influenced by the physiological setting in which these events take place. In this study, we explored the surface chemistry of HIV-1 Env constructs at a range of pH and salinities relevant to mucosal and systemic compartments through electrophoretic mobility (EM) measurements. Sexual transmission events provide a more acidic environment for HIV-1 compared to dissemination and spread of infection occurring in blood or lymph node. We hypothesize functional, trimeric Env behaves differently than monomeric forms.

**Results:**

The dynamic electrophoretic fingerprint of trimeric gp140 revealed a change in EM from strongly negative to strongly positive as pH increased from that of the lower female genital tract (pHx) to that of the blood (pHy). Similar findings were observed using a trimeric influenza Haemagglutinin (HA) glycoprotein, indicating that this may be a general attribute of trimeric viral envelope glycoproteins. These findings were supported by computationally modeling the surface charge of various gp120 and HA crystal structures. To identify the behavior of the infectious agent and its target cells, EM measurements were made on purified whole HIV-1 virions and primary T-lymphocytes. Viral particles had a largely negative surface charge, and lacked the regions of positivity near neutral pH that were observed with trimeric Env. T cells changed their surface chemistry as a function of activation state, becoming more negative over a wider range of pH after activation. Soluble recombinant CD4 (sCD4) was found to be positively charged under a wide range of conditions. Binding studies between sCD4 and gp140 show that the affinity of CD4-gp140 interactions depends on pH.

**Conclusions:**

Taken together, these findings allow a more complete model of the electrochemical forces involved in HIV-1 Env functionality. These results indicate that the influence of the localized environment on the interactions of HIV with target cells are more pronounced than previously appreciated. There is differential chemistry of trimeric, but not monomeric, Env under conditions which mimic the mucosa compared to those found systemically. This should be taken into consideration during design of immunogens which targets virus at mucosal portals of entry.

## Background

Characterization of the envelope glycoprotein (Env) on HIV-1 has been a focus of vaccine development, both for mechanistic and immunogen design purposes. Understanding the conformational properties of Env under physiologically relevant conditions may be critical to effective targeting of antibodies and antiviral agents. The experimental conditions typically used for study of HIV-1 binding and entry are not highly representative of the mucosal environment, which has a wide range of characteristics not seen systemically, particularly with regard to pH. The Env protein is transcribed as a 160 kDa glycoprotein, which is subsequently cleaved through a cellular protease to yield a membrane spanning subunit, gp41 and a non-covalently linked surface protein, gp120 [1-3]. Functional envelope spikes are made of trimeric heterodimers of these subunits and range in number from 7 to 14 over the surface of a viral envelope from primary isolates of HIV-1 [4,5].

The gp120 molecule is comprised of an inner domain and a highly glycosylated outer domain, connected by a bridging sheet [6]. The inner domain of the gp120 molecule was recently shown to be composed of a 7 stranded β- sandwich and a series of flexible semi-structured loops, from which emanate the variable regions 1–3 [7]. These domains have a high degree of plasticity and substantial entropy in their unliganded state, which is partially used to stabilize the ligand-bound state [8-10]. The high potential energy is used to overcome the energetic barriers to fusion of the viral and target cell membranes [7]. The primary receptor for HIV-1 Env is the CD4 molecule which binds through a conserved surface on the gp120 outer domain [11,12]. Following initial CD4 engagement, gp120 undergoes a decrease in structural entropy that enables stable CD4 recognition and binding [9]. Post binding, three discrete events occur: an outward density shift, a gp120 tilt away from the central z-axis, and a gp120 rotation which flattens the trimer ostensibly allowing the fusion peptide to protrude and insert into the host membrane [13]. These changes to gp120 structure promote interaction with one of two chemokine receptors, either CCR5 or CXCR4 (R5- and X4-, respectively) [14-17] triggering extensive conformational rearrangement in gp41 facilitating subsequent membrane fusion [18,19].

The majority of immunization studies with HIV-1 Env have used gp120 glycoprotein and have shown poor induction of broadly neutralizing antibodies against clinically-relevant viral strains [20]. This may be due to the structural flexibility of the monomer or conformational differences compared to the functional envelope-bound trimer. Immunization with soluble trimeric glycoprotein is therefore considered an improvement upon the gp120 model. There have been a number of biochemical efforts to describe the structure of the native Env trimer that reveal a degree of plasticity in higher order structure [7]. Within trimers the variable loops of gp120 take on multiple conformations dependent on receptor and co-receptor binding [21], and there exist multiple interactions between subunits [22-24]. Multiple open and closed conformations are continuously sampled within monomers, without one fixed set of epitopes exposed [21,25,26]. However, recombinant trimeric gp140, while more physiologically relevant than the gp120 monomer, differs from native trimers in that the natural cleavage site between gp120 and gp41 is usually mutated and only the ectodomains of gp41 with the trimerization motif are included. Nevertheless, glycosylation patterns are unchanged between the native envelope and the recombinant protein, as is the trimeric configuration [27,28]. The exposure of the CD4 binding site and its binding behavior relative to whole virions is seen to be preserved in recombinant trimeric proteins [26-28].

High affinity molecular interactions, such as the binding of gp120 to CD4, are dictated by surface charge and the relative hydrophobicity of complementary binding sites of ligand pairs as well as the net entropic and enthalpic states. Stable binding events present a specific binding site of one molecule that uniquely partners a receptor of complementary charge. Interactions are also influenced by the isoelectric point (IEP) of each amino acid that comprises the binding site. Nondestructive electrophoretic mobility (EM) measurements allow the characterization of surface charge over a wide range of pH and electrolyte concentration. Characterization of cells by their electrophoretic properties has allowed new insights into cell-cell interactions [29]. The process of dynamic electrophoretic fingerprinting (DEF) involves plotting the surface charge, expressed as EM, over a physiologically relevant range of pH and salinities (expressed as pλ, the negative log transform of solution conductivity). EM is a scaled factor of ζ-potential which is also frequently used to describe surface chemistry due to its convenient units of mV. The conditions under which a protein attains a net-zero EM is known as its isoelectric point (IEP), and the continuous line of pH/pλ conditions where a surface is at its isoelectric point is termed the line of zero mobility (LZM). This technique can give insights into the chemical composition of a surface: slope indicates the rate and difficulty of protonation; IEP indicates the balance of charged moieties; pH or salinity induced structural changes can be inferred from sharp changes in surface potential [30-33]. While the entry of HIV-1 into cells is not dependent on pH, there are a number of studies which have shown non-classical entry mechanisms for virions [34-36].

To our knowledge, while cells and micro-organisms have been characterized and separated by their electrophoretic mobility [29,35,37] this technique has never been applied to the characterization of virions or their envelope glycoproteins. We hypothesize that the environment and oligomeric state of Env will influence its conformation and binding properties. As HIV contains a range of non-virally encoded host proteins in addition to the envelope spike, to isolate the effect of the Env protein from total virion surface chemistry, measurements presented in this study were initially made using clade B gp120 monomers, as well as clade B and C gp140 trimeric Env. These measurements are supported by computational modeling of gp120 crystal structures across pH. However, the titration technique used in DEF allows additional modeling of the charge characteristics and by implication altered conformational states not discernable with fixed crystal models. We have modeled these changes across salinities and pH ranges relevant to the physiological environments associated with mucosal transmission and systemic infection. DEF of the trimer were contrasted with that of whole virions and those of resting and activated CD4^+^ T cells. The data presented provide an electrochemical model of the forces affecting HIV-1 Env in the context of mucosal and systemic environments, and illustrate the important differences in the conformational dynamics specific to trimeric Env.

## Results

### Dynamic electrophoretic fingerprint of monomeric BX08 gp120

**Figure 1:**
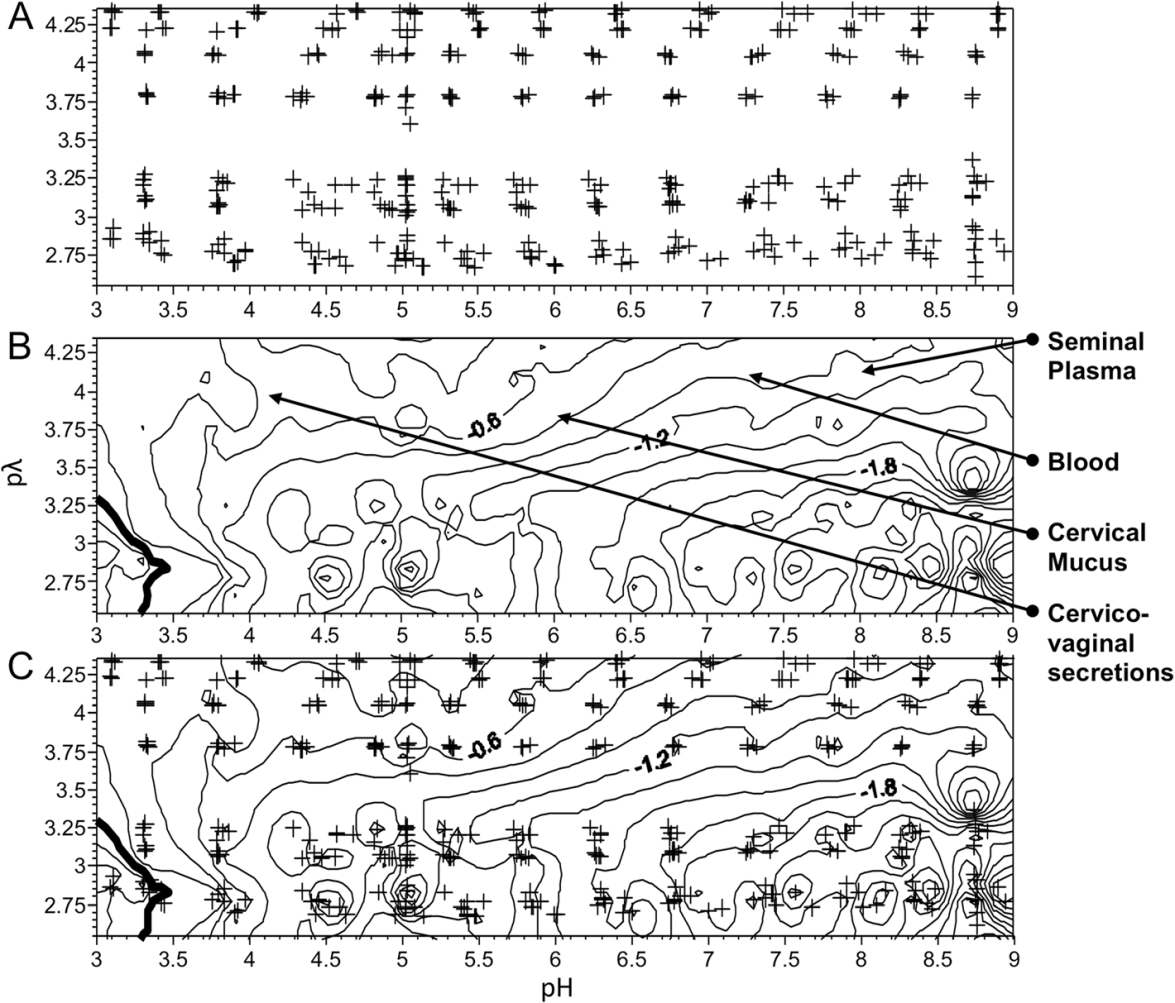
**Dynamic electrophoretic fingerprint (DEF) of monomeric BX08 gp120.** Mobility measurements were made across a pH titration (3.0-9.0) in 0.5 pH increments) and over a range of salinities (1-200 mM NaCl), expressed as the negative log of the conductivity (pλ 2.60 to 4.28). Titrations were performed in triplicate, with three measurements made at each pH for all pλ conditions. (**A**) Postage stamp plot showing distribution of data collection. Each cross represents the average pH and pλ of three electrophoretic mobility measurements. (**B**) Contour plot of the electrophoretic fingerprint generated from the data collected in (**A**). Line of zero mobility (LZM), bolded, indicates the isoelectric point at the lowest pH and pλ conditions examined. Bold black lines indicate mobility changes of 0.6 μmcm/Vs. Arrows indicate the pH and pλ conditions of several relevant biological mediums. (**C**) Overlay of postage stamp and DEF shows data distribution includes all key features.

Initial studies were performed to determine the electrophoretic mobility (EM) of recombinant monomeric gp120 derived from the clade B HIV-1 strain BX08. This generated 1071 data points that were averaged at each titration point and are represented as a postage stamp map (Figure 1A). These data were used to generate a dynamic electrophoretic fingerprint (DEF) of measurements corresponding to each pλ and pH value plotted as a series of levels showing change in surface charge over the range of conditions (Figure 1B; Table 1 “BX08 gp120”).

**Table 1:** Electrophoretic mobility (EM) of HIV and Influenza envelope protein constructs

**Electrophoretic mobility of viral envelope proteins at two salinities (μmcm/Vs)**								
**pH**	**BX08 gp120**		**BX08 gp140**		**CN54 gp140**		**H3N2 HA**	
	**2.75**	**4.15**	**2.75**	**4.15**	**2.75**	**4.15**	**2.75**	**4.15**
4.5	−0.21	−0.44	0.23	0.00	−0.22	−0.60	ND	−1.47
5.5	−1.09	−0.41	−0.47	−0.27	−0.63	−0.81	ND	−1.56
6.5	−1.40	−0.80	0.53	0.13	−0.40	−0.60	ND	−1.04
7.5	−2.34	−0.94	0.36	0.69	0.44	−0.63	ND	1.44
8.5	−2.27	−1.15	−0.01	0.17	−0.88	−0.79	ND	0.26

EM measurements were made for each protein at a range of pH and pλ conditions; a subset of the observed mobilities is shown here. ND = not determined.

**Figure 2:**
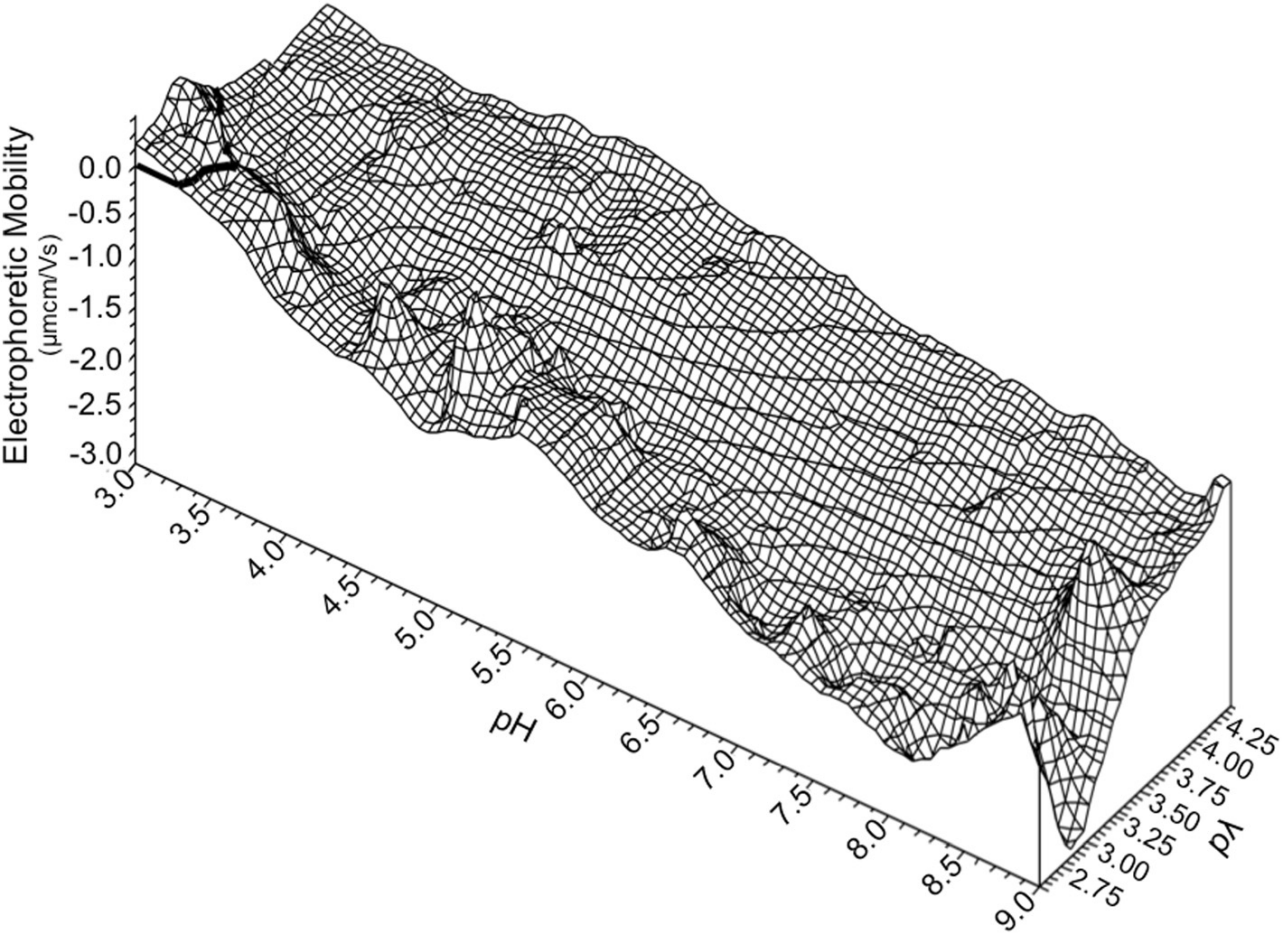
**BX08 gp120 monomer dynamic electrophoretic fingerprint (DEF) shown as a three dimensional wireframe model.** Bolded line represents zero electrophoretic mobility (LZM). A single LZM is observed in the most acidic and lowest conductivity conditions, Mobility is less than zero under nearly all pH/pλ combinations. The fingerprint reaches a minimum of −2.4 μmcm/Vs at the most alkaline. The DEF exhibits the twisted ribbon shape seen with other biological surfaces. Data were plotted using direct linear interpolation.

These data exhibit several key features. The general shape of the DEF plot resembles that of a “twisted ribbon” (Figure 2). For a given pλ as the pH increases from low to high, there is a decrease in EM. Increases in pλ have a buffering capacity on EM whereby in a high pλ environment (top of graph 1B), EM is closer to zero than at lower pλ (bottom of graph 1B). Higher salinities show less change in EM during a titration while lower salinities exhibit a more pronounced change in mobility, due to the increase in exposure of glycoprotein associated charged species when there are fewer solution ions to mask the effect. Negative mobility measurements were observed over nearly all conditions for BX08 gp120, with the exception of the most acidic and the lowest salinity - pH less than 3.5 and pλ below 3.25. This indicates a principally anionic charge to the protein, even under conditions of significant acidity. The line of zero mobility (LZM), bolded, shows the conditions under which the EM changes from positive to negative, and was largely absent over the conditions examined.

The spread of the data (Figure 1A), when compared to the electrophoretic fingerprint (Figure 1B), indicates that the frequency and distribution of sampling was appropriate. Overlaying the postage stamp map with the topographic map (Figure 1C) shows sampling of EM is evenly distributed throughout the pH and pλ ranges of interest. The mobility lines all trace the twisted ribbon shape and include the key features of decreasing mobility as pH is increased, and increasing amplitude as salinity decreased.

Plotting the DEF on a three dimensional wireframe illustrates these features more clearly (Figure 2). The most notable fluctuations occur at the low salinity measurements of pλ of 2.8 (1.0 - 5.0 mM NaCl). The pH and salinity conditions that correspond to those of vaginal fluid, cervical mucus, seminal plasma, and blood plasma (indicated on Figure 1B), all display a negative charge for the BX08 gp120 protein.

### The electrophoretic fingerprint is consistent with modeled surface potential of gp120 monomer crystal structures

Several structures have been determined for gp120 monomer of HIV-1 using X-ray crystallography. However, there is no high-resolution structure of entire native gp120 monomer. In all available structures, HIV-1 gp120 is either in complex with its natural ligand, CD4, or an antibody. Also, they lack the variable regions such as V1/V2 loops and N- and C-terminal regions that interact with gp41. An unliganded gp120 core is available for an SIV strain also lacking in V1/V2 and V3 loops and terminal regions. Also, it has been recently reported that this gp120 core conformation from SIV does not lead to an optimal fit with the cryo-electron microscopy density maps of liganded and unliganded HIV-1 gp120 trimers on the viral membrane [38]. Given these limitations, we undertook electrostatic surface potential (ESP) calculations on a series of gp120 monomer structures as described below, their comparison providing a more comprehensive picture than that of any individual structure. ESP was performed across a range of pH and pλ values to correlate with the DEF measurements. ESP calculations were performed by numerically solving the full nonlinear form of the Poisson-Boltzmann equation using the APBS software v1.4 [39] at a temperature of 310 K with standard parameters (see methods for details).

**Figure 3:**
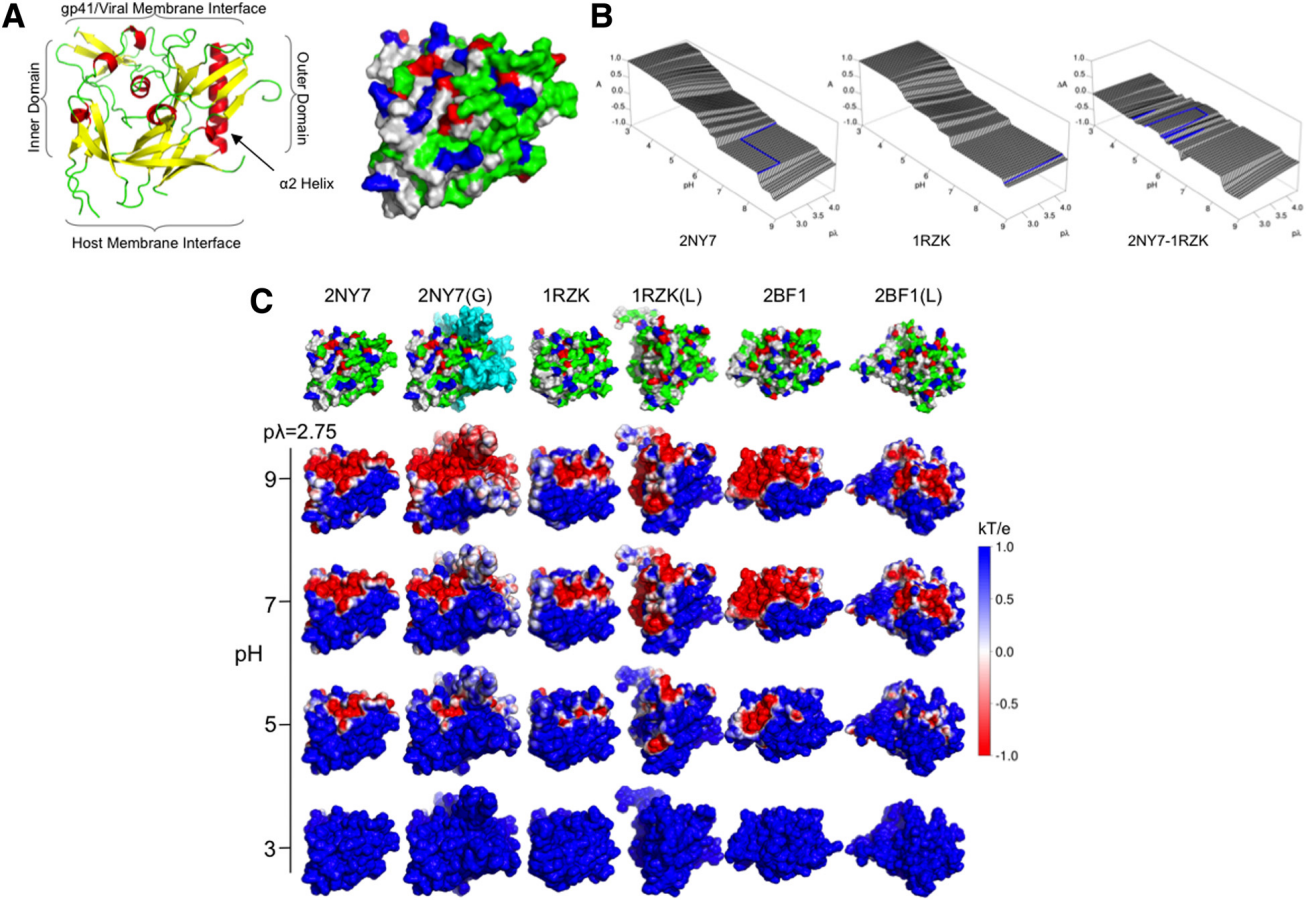
**Computational models of electrostatic surface potential on multiple forms of gp120.** (**A**) Ribbon and space-filling models of gp120 core construct (PDB code 2NY7). (**B**) Electrostatic potential over a range of pH/pλ conditions of b12-bound core and CD4 and 17b bound core gp120 constructs, and the difference between structures. (**C**) Space-filling surface maps indicate the local changes surface potential of 6 gp120 constructs across pH, ranging from fully protonated (blue) to fully deprotonated (red).

First, we consider the unglycosylated gp120 core structure bound to the antibody b12 (PDB code 2NY7) [12], a three-dimensional structure of this gp120 core with key regions marked is shown (Figure 3A). We based our primary analysis on this structure and used additional structures to illustrate the effects due to ligands, loops and glycosylations. The ESP for 2NY7 is shown in Figure 3B. These data indicate that the twisted ribbon shape seen in the DEF of BX08 gp120 is representative of the Env monomer. Lack of strict quantitative agreement to measurements can be attributed to the fact this structure is not able to adopt the range of conformations available to a native gp120 monomer in solution, but is restricted to effects on a fixed solvent exposed surface.

Next, we considered three different structures; 2NY7(G), 1RZK, and 1RZK(L). 2NY7(G) is a modified form of the b12 bound gp120 core where N-glycosylations have been modeled by adding five-mannose glycans, found to be the most abundant glycan forms in viral envelope [40]. Electrostatic surface potentials of these structures were compared against the base structure (2NY7) (Figure 3C; Additional file 1: Figure 1 and Additional file 2: Figure 2). The addition of high mannose had the net effect of increasing the surface potential across all pλ values in the pH range between 4 and 8.

To represent a CD4-bound gp120 core, we considered the X-ray structure where gp120 core was crystallized with CD4 and 17B antibody (PDB code 1RZK). The surface potential in the CD4 binding site of 1RZK complements the surface potential of CD4 receptor. Changes in gp120 core upon binding to CD4 are found in the acidic (pH 3.5-5.0) and lower salinity conditions and result in a more positive surface potential than the b12 bound form (Figure 3C). 1RZK (L) is a CD4-bound gp120 structure where all variable loops and N- and C-terminal regions have been modeled. A long time scale all-atom molecular dynamics simulation randomly selected this configuration. This simulated gp120 is unglycosylated and modeled using all known liganded gp120 structures. In the simulations, 1RZK (L) core did not undergo large conformational changes from the initial CD4 bound configuration of 1RZK. Detailed descriptions of the above modeling approaches are described elsewhere [41]. The influence of including previously missing domains is to increase the surface potential at the low pH ranges as seen for 1RZK, as well as decrease it in the pH range 5.0 to 6.5. These effects are shown by comparing the base structure (2NY7) to 1RZK or 1RZK (L) (Additional file 3: Figure 3 and Additional file 4: Figure 4).

Given that the native (unliganded) HIV-1 gp120 monomer remains unresolved, we considered two additional monomer constructs from SIV for comparison with our base structure [22], the first being the gp120 core crystal structure (PDB code 2BF1). The second, 2BF1 (L), includes modeling of the loops and terminal regions in a similar fashion to 1RZK(L). 2BF1 has a more positive surface potential than the 2NY7 in the pH range from 4.0 to 5.0, and is more negative above pH 6 (Additional file 3: Figure 3 and Additional file 4: Figure 4). Comparison of the 2BF1(L) and 1RZK(L) structures helps illustrate the effect of CD4 on surface charge exposure (Additional file 5: Figure 5, Additional file 6: Figure 6 and Additional file 7: Figure 7). CD4 binding results in an increased exposure of positively charged moieties in the outer domain, while both structures including variable loops are more negatively charged at neutral and alkaline pH (Figure 3C, Additional file 7: Figure 7). Comparison of all the space-filling models of these surfaces at pH values between 3 and 9 indicates site specific deprotonation rather than uniform change in surface potential as a function of pH (Figure 3, Additional file 7: Figure 7). The host-membrane interface region retains a positive charge under a wide range of conditions. A systematic comparison of all gp120 monomer constructs to DEF measurements suggest that the electrostatic surface potential of a native gp120 monomer is greatly influenced by the placement of variable loops and is more pronounced in liganded gp120 monomer structures. This difference, due to inclusion of variable loops, implies strain-dependent influences on surface potential, at least within monomeric gp120.

### Dynamic electrophoretic fingerprint of BX08 gp140 trimer displays conformational change not seen on gp120

**Figure 4:**
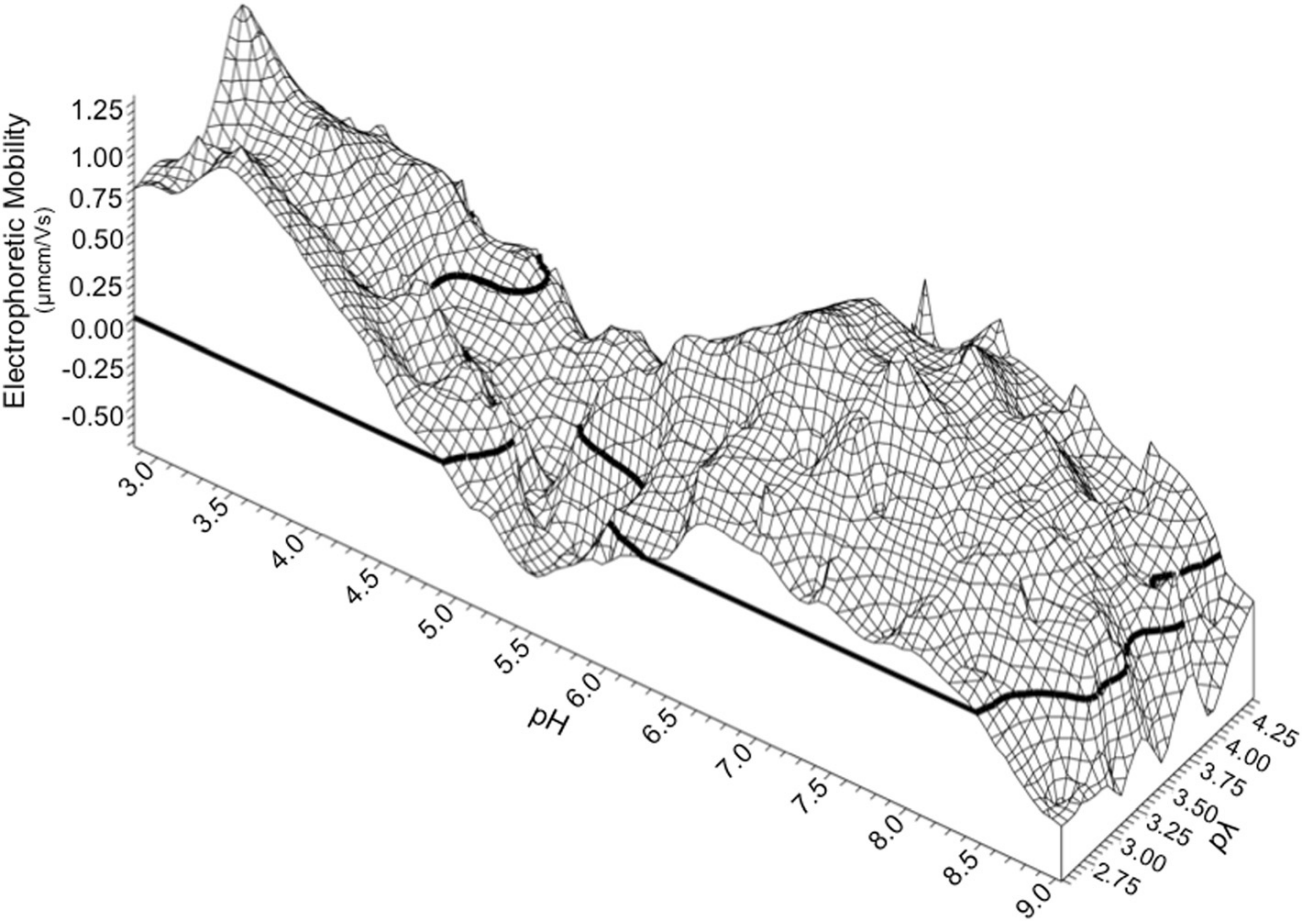
**BX08 gp140 trimer dynamic electrophoretic fingerprint (DEF) shown as a three dimensional wireframe model.** Bolded lines show the line of zero electrophoretic mobility (LZM). There are three LZMs seen in the DEF: first at pH 4.9 and pλ 2.55 which curves left at higher pλ conditions; a second LZM is seen at a pH of 5.5 to 6.5 after which electrophoretic mobility increases to a maximum of 1.2 μmcm/Vs at pH 6.8-7.5 and pλ of 3.25; and a third LZM is seen at pH 8.5 above which mobility is negative. Data were plotted using direct linear interpolation.

Gp120 monomer in a functional trimer is expected take on a different conformation as constrained by the packing of variable loops of oligomeric units and the gp41. Further experiments were performed to generate a DEF of trimeric BX08 gp140 (Figure 4). Each gp140 monomer comprises the entire gp120 protein and the gp41 ectodomain, giving a more complete model of the functional Env complex, and the material studied is primarily comprised of trimeric protein. The data distribution collected from a series of titrations was uniform and well distributed over the area under examination (Additional file 8: Figure 8A). A topographical plot of the DEF shows significant differences to that seen with gp120 (Additional file 8: Figure 8B; Table 1 “BX08 gp140”). Strikingly, trimeric BX08 gp140 displayed islands of positivity that correspond to the solution conditions of serum, unlike gp120 that was predominantly negative over all conditions. As the protein was titrated from pH 3.0 upwards the mobility initially decreased, as expected with the addition of anions. The DEF reached a minimum in the region spanning pH 5.2 to 5.5, after which there was a steep increase in mobility (slope >2.0 μmcm/Vs per pH unit) until a local maxima was attained. This resulted in a second LZM being present between pH 5.5 and 6.7 with a ridge forming around the median pλ values. After reaching a maximal positive mobility near neutral pH, the addition of further anions decreased mobility. The decrease seen above pH 7.0 to 7.5 was not as steep as the increase near pH 5.5, but had a similar gradient profile to the titration at low pH. At the most alkaline conditions examined, the mobility again became negative. At all ranges of pλ, the titration between pH 5.5 and 9.0 were quite similar, in contrast to the gp120 measurements, which had more charge exposure in low conductivity environments and the twisted-ribbon characteristic.

The three dimensional wireframe map of trimeric BX08 illustrates the dynamic fluctuations in charge polarity for trimeric gp140 over pH, compared to the steady decrease in mobility observed for the monomeric protein. While local fluctuations in mobility were observed, these are indicative of the uncertainty of the measurement rather than true localized changes. Such variability indicates that the EM of the trimer is not rigid, and is able to adopt a narrow range of electrophoretic mobility values in any pH and pλ environment. The major switch in the polarity of mobility (from negative to positive), counter to the charge of the titrant, indicates that more than a simple protonation-deprotonation equilibrium shift is occurring. Mobility changes counter to ion charge are indicative of a structural rearrangement [33]. Thus the increase in EM from a pH of 5.5 to 7.0 likely corresponds to exposure of positively charged amino acids which had previously been shielded, such as the cross-clade conserved (Arg-Pro) and (Gly-Pro-Gly-Arg) motifs used for coreceptor interaction in the V3 loop [42].

### Dynamic electrophoretic fingerprint of clade C CN54 gp140 trimer exhibits similar features to clade B BX08 gp140

**Figure 5:**
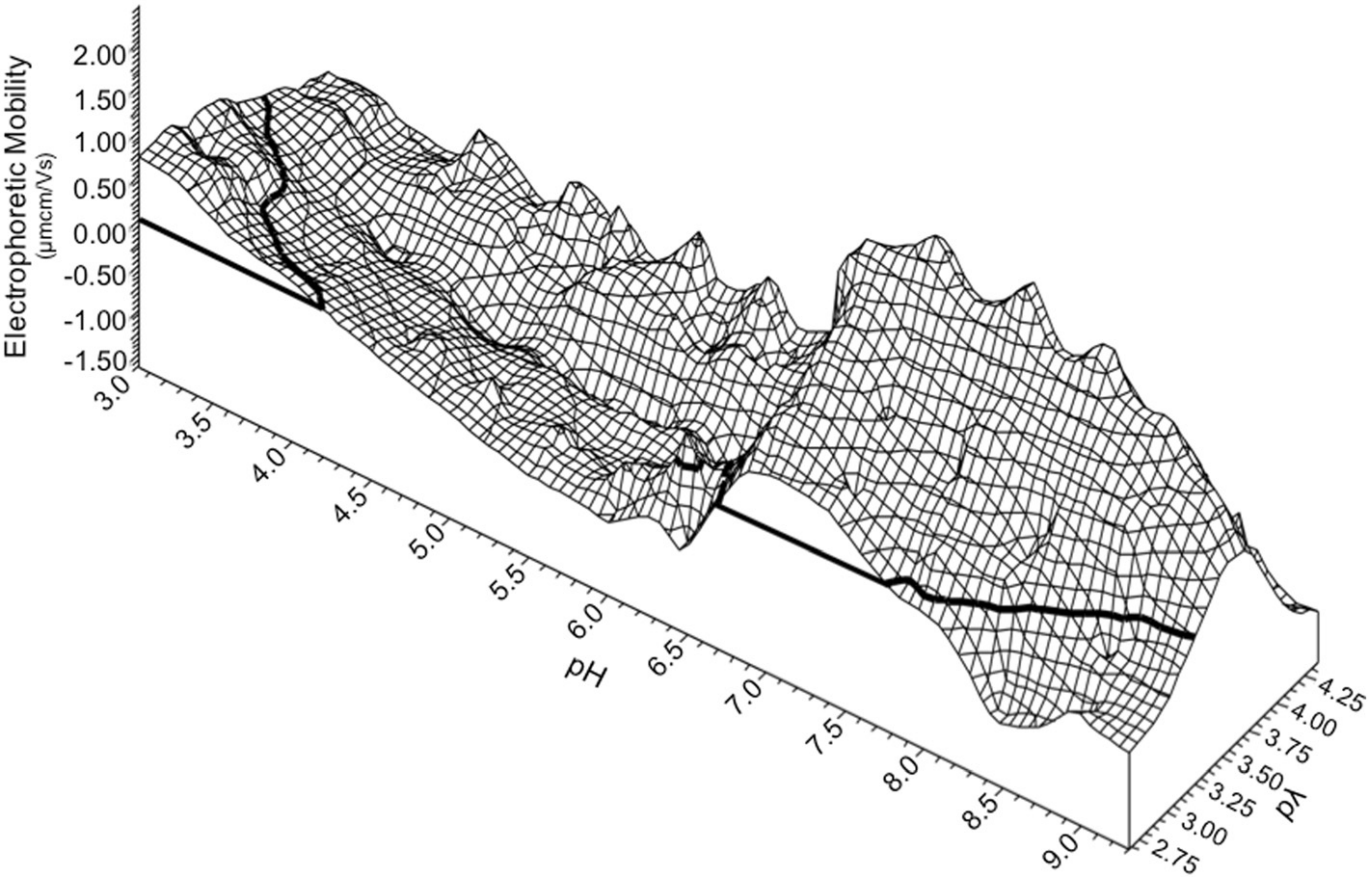
**CN54 gp140 trimer dynamic electrophoretic fingerprint (DEF) shown as a three dimensional wireframe model.** Bolded lines show the line of zero electrophoretic mobility (LZM). There are three LZMs seen in the DEF: first at pH 4.2 and pλ 2.55 which moves to the left at higher pλ conditions. A second LZM is seen at a pH of 6.7 at low conductivity, which becomes horizontal at pλ above 3.85, after which electrophoretic mobility increases to 2.0 μmcm/Vs at pH 7.4-8.0 and pλ of 3.62. A third LZM is seen at pH 7.7 and low conductivity which moves to the right at higher conductivity and beyond which mobility is negative. Data were plotted using direct linear interpolation.

In order to compare the envelope trimers from clade B, the most prevalent viral clade in Europe and North America, with those of clade C, the clade most prevalent across Sub-Saharan Africa, measurements of CN54 gp140 were made (Figure 5, Additional file 9: Figure 9). This protein is also almost exclusively trimeric and remains stable over long timescales [43]. As in BX08 gp140, this construct contains the entire gp120 protein and the gp41 ectodomain with trimerization motifs. The DEF displayed similar behavior to that of the clade B trimer (Figure 5; Table 1 “CN54 gp140”). Three LZMs are observed highlighted by bold contours. The leftmost LZM seen at low pH had an IEP of 4.2, which moved to the left in higher salinities. The electrophoretic mobility of the protein then decreased steadily until the global minimum was reached at pH of approximately 5.5. After this, a rapid increase was observed, with a second LZM seen at pH 6.7. The DEF reaches a positive maximum at pH 7.4 of 2.40 μmcm/Vs. For the clade C envelope, the positive behavior was not observed at the highest pλ values; instead the LZM became horizontal from pH 6.8 through the highest pH values examined. Orthogonal to the horizontal LZM, electrophoretic mobility sharply increased with decreasing pλ, reaching a maximum at a pλ of 3.63, after which the mobility decreased as salinity continued to decrease. This horizontal isoelectric region was just below a pλ of 4.0 (100 mM NaCl). The data distribution in the region of the DEF surrounding the horizontal portion of the LZM is less dense. This region, between pλ of 3.68 and 3.94, corresponds to salinities between 50 and 100 mM NaCl, (Additional file 9: Figure 9C) and does not appear to exclude features of the DEF. The mobilities at higher and lower salinities than the LZM reflect a sharp trend in EM, with data points sampling the negative, zero and positive EM environments. A third LZM is observed in the bottom right section of the DEF, where low pλ and high anionic conditions drive the protein into a deprotonated state. This LZM originates at a pH of 7.72 and pλ 2.68, and moves sharply to the right at higher pH. As pλ increases from 3.25 up to 3.75 (10-50 mM NaCl) the protein surface never reaches an IEP under the pH conditions examined, indicating surface groups extremely resistant to deprotonation.

The DEF shows the general features to be very similar between the clade B and clade C gp140 trimers. The nonlinear nature of the gp140 fingerprints compared to that of gp120 agrees with the structural descriptions of Env proteins in that there were a range of conformations and epitopes exposed across physiologically relevant pH and pλ conditions.

### HIV-1 Env constructs from chronic and transmitted strains have similar electrophoretic mobility

**Figure 6:**
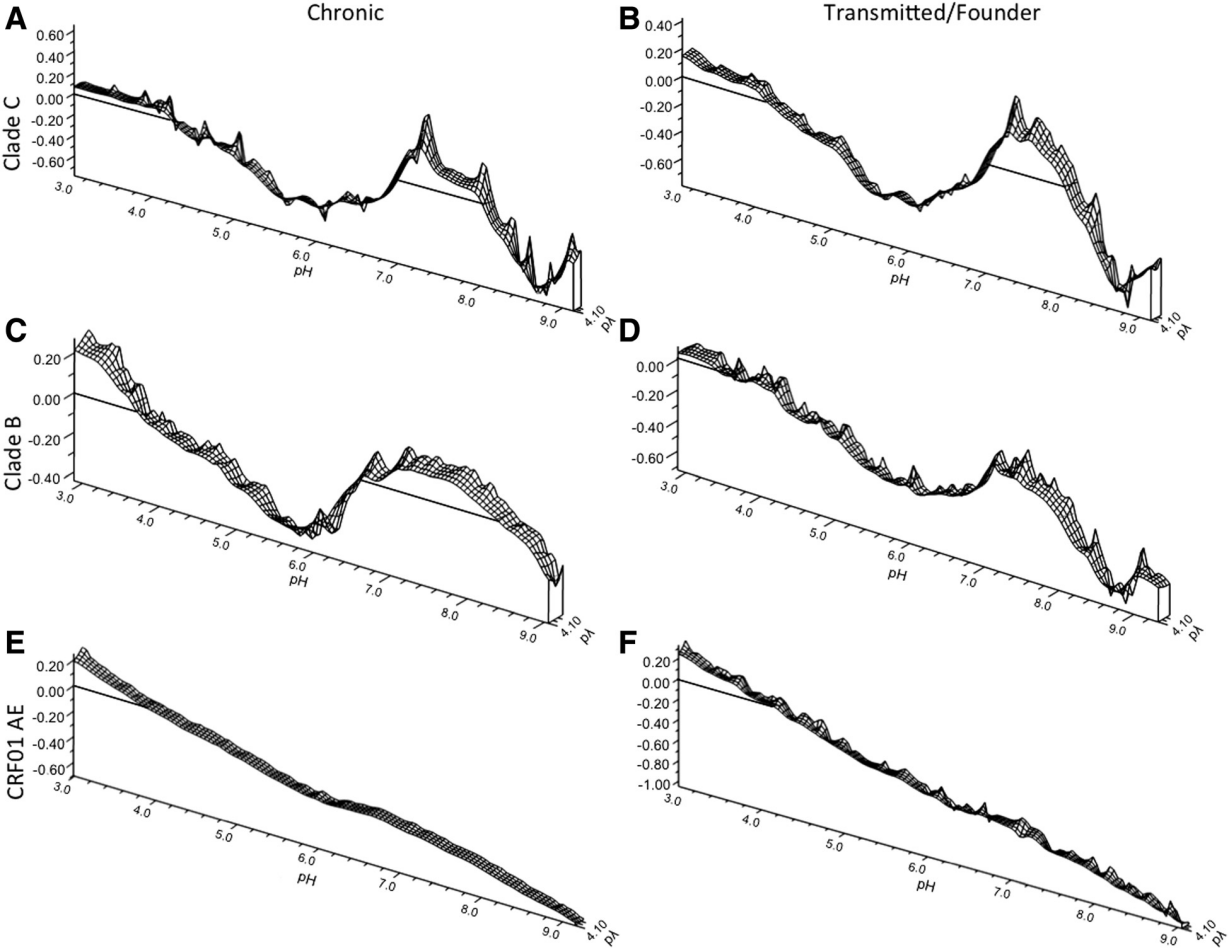
**Partial dynamic electrophoretic fingerprint of monomeric and trimeric viral envelopes from multiple clades with diverse virological phenotypes.** Electrophoretic mobility (EM) measurements were made for three chronic viral strains from (**A**) clade C (DU123 gp140), (**B**) B (JRFL gp140) and (**E**) CRF01_AE (A244 Δ11 monomer) and three transmitted/founder viral strains from (**B**) clade C (1086C gp140), (**D**) clade B (63521 gp140) and (**F**) CRF01_AE (RV144 427299Δ11 monomer). Horizontal lines indicate the line of zero electrophoretic mobility Data was collected for three titrations, and three measurements were made and averaged at each titration point (n = 9 per titration point). Data were plotted using direct linear interpolation.

In order to make these results more generalizable to primary isolates from diverse HIV strains, additional mobility measurements were made for 6 envelopes, with transmitted/founder (T/F) strains and chronic strains from clade B, C and CRF01 (Figure 6). Collectively, these data indicate that it is the trimeric structure of Env proteins which gives rise to the islands of positivity seen near neutral pH. There is a shared profile amongst the 6 trimeric HIV-1 Env proteins that is distinct from the three monomeric forms. Viral envelopes from clade B or C in their trimeric forms have similar profiles whether they are of T/F or chronic phenotype (Figure 6A-D). Strain DU123 is resistant to antibody neutralization [44], but the DEF mirrors the shape of neutralization sensitive JR-FL. The T/F strain 63521 gp140 has only limited positivity to its DEF in the region of neutral pH. Within CRF01, only monomeric gp120 proteins were available to us, and these behaved as seen with BX08 gp120 and modeled ESP measurements, varying only in the magnitude of the negative mobility they could adopt under alkaline conditions. The profile of both viral strains is uniformly negatively sloped under the full range of pH.

The magnitude of strain dependent differences is less significant between the trimeric Env relative to the monomer, indicating that the variable loops are packed between monomers and at least partially concealed from the solvent layer. These data indicate that the primary source of variation between DEFs of distinct viral Env strains is the solvent exposed variable domains. The protein surface undergoes a titration process which is dependent on the oligomeric state of Env, with relative changes in the magnitude of EM determined by strain specific amino acid differences.

### Trimeric influenza Hemagglutinin exhibits parallel inflections to those observed with HIV-1 Env

To determine whether the dynamic nature of the gp140 trimer was reflective of trimeric envelope proteins from other viruses, studies were performed using the trimeric form of the hemagglutinin protein (HA) of influenza (strain H3N2). The binding and fusion events of the HA complex on influenza virions is probably the most completely studied viral membrane fusion system [45,46]. The trimeric heterodimer of HA1 and HA2 molecules undergoes many folding events which are analogous to the gp41/gp120 complex binding and mediating viral fusion with target cells. Unlike HIV-1, HA mediated influenza fusion with, and entry into target cells is triggered by a decrease in pH during acidification of endosomes. Under acidic conditions HA undergoes a large scale conformational rearrangement which results in the formation of an “extended intermediate” structure from which membrane fusion progresses.

**Figure 7:**
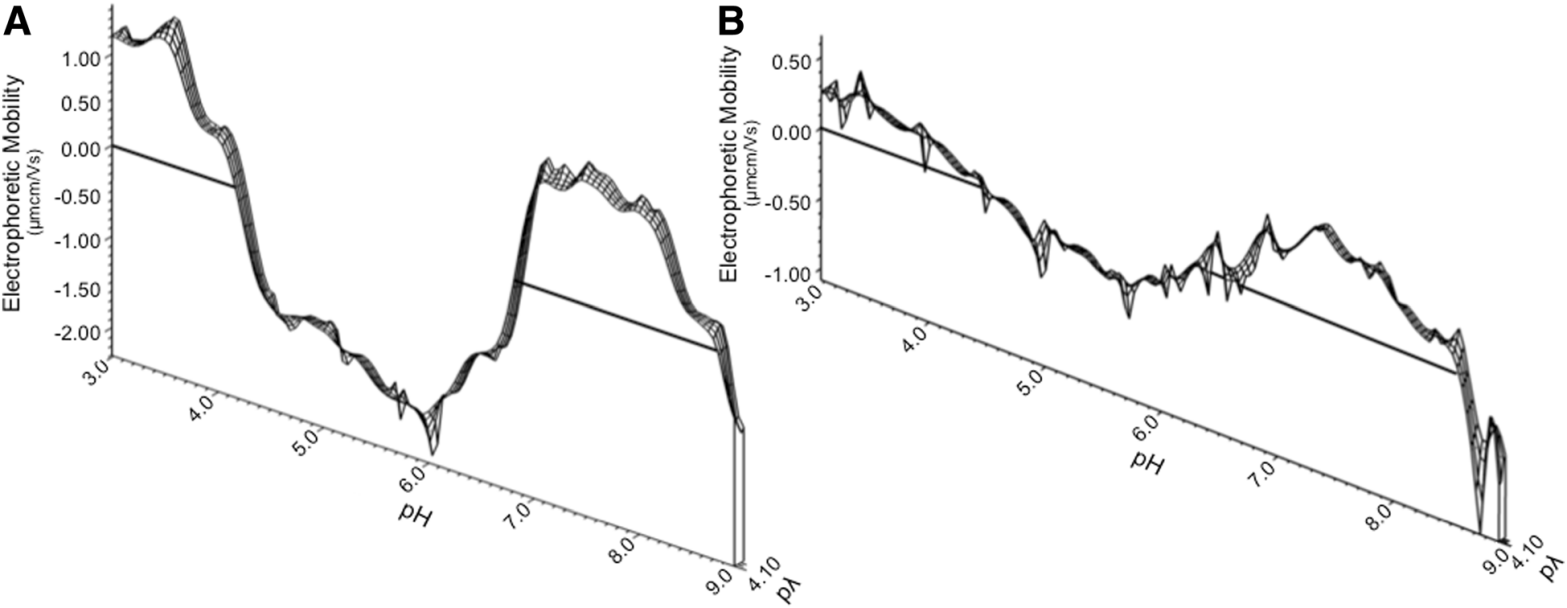
**Partial dynamic electrophoretic fingerprint of trimeric (A) influenza HA and (B) HIV-1 BX08 gp140.** Electrophoretic mobility was measured at 13 pH values ranging from 3.0 to 9.0 in 154 mM NaCl. Bolded lines show the line of zero electrophoretic mobility. Data was collected for three titrations, and three measurements were made and averaged at each titration point (n = 9 per titration point). Data were plotted using direct linear interpolation.

To compare the DEF of trimeric HA to that of trimeric gp140, measurements were made over the same broad pH range (Figure 7A, Table 1 “H3N2 HA”). Limited material availability restricted the measurements to a physiological salinity, corresponding to a pλ of 4.1-4.2. At the highest hydrogen ion concentrations positive electrophoretic mobility was observed, which continued to decrease until pH 5.65, with an IEP at pH 4.08. EM decreased until pH 6.1, where the minimum observed mobility was recorded. As pH further increased to neutral, electrophoretic mobility returned to positivity. A maximal ζ-potential was recorded at pH 7.58 which is similarly positive (1.41 μmcm/Vs) to the behavior measured at the most acidic pH examined (1.21 μmcm/Vs). Above pH 7.58, electrophoretic mobility decreased sharply and became negative under the most anionic conditions, near pH 9.0. This profile, with increasing surface potential between pH 5.65 and 7.25, was similar to those seen with CN54 and BX08 gp140 trimeric Env in the same environment. The magnitude of the surface potential of trimeric HA was greater than that of the Env proteins in both the positive and negative directions, but the IEPs and curve shape were analogous. Direct comparison to the BX08 gp140 trimer is possible by examining a slice of EM data taken under similar salinity conditions (Figure 7B). There is a high degree of homology between the titrations: three IEPs are observed in each and the curves display the same shape over the broad pH range. Deviation from zero EM is more pronounced in the HA trimer than Env but localized fluctuations in each curve are between 0.1 and 0.2 μmcm/Vs. While similar structural rearrangements may be occurring in each, the number and degree of charged moieties that become alternately exposed or shielded varies between the viruses. These results indicate that the findings seen with HIV Env glycoproteins may be a generalized attribute of enveloped viral glycoproteins and not specific to the HIV system.

### Modeling surface charge effects of pH induced triggering of HA

**Figure 8:**
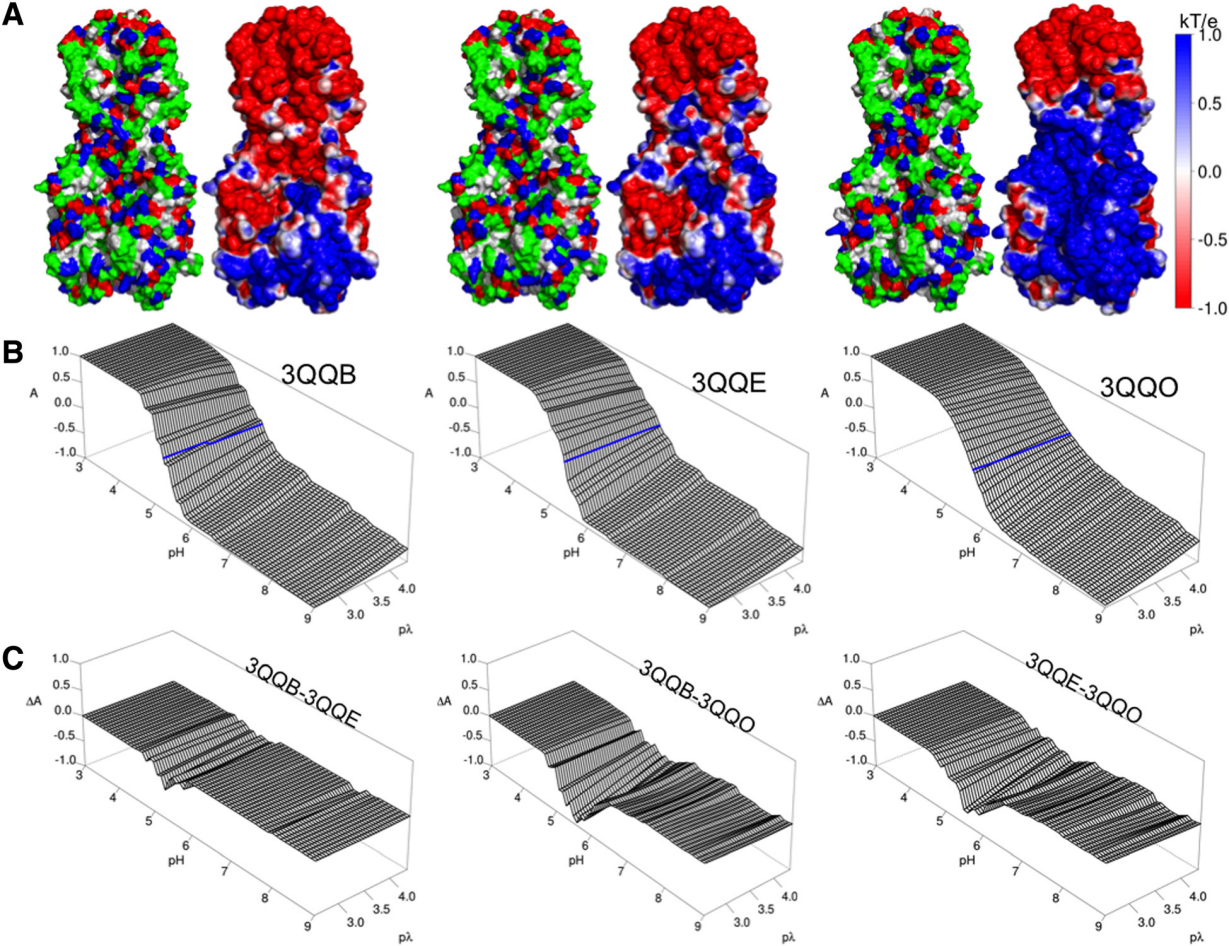
**Electrostatic surface potential calculations on differing conformational states of trimeric influenza HA protein.** (**A**) The amino acid type (left panels) and electrostatic surface potential (right panels) of 3 HA protein forms – native protein conformation, low pH triggered conformation after being reneutralized, and low pH conformation (PDB codes 3QQB, 3QQE and 3QQO respectively) all shown under pH 5.5 conditions. (**B**) Electrostatic surface potential charge asymmetry (**A**) over a range of pH/pλ shows the relative degree of protonation for each HA structure. (**C**) Differences in charge asymmetry (ΔA) between HA conformations are focused around pH 5.5.

The structure of HA has been resolved at the molecular level under various solution conditions that reveal differences between native and triggered forms of the complex. This allows comparisons between the DEF data and known conformations adopted by trimeric viral envelope proteins, here modeled as native HA (PDB Code 3QQB), pH triggered HA (PDB Code 3QQO), and pH triggered HA after it has been re-neutralized (PDB Code 3QQE) (Figure 8A; Additional file 10: Figure 10 and Additional file 11: Figure 11). ESP calculations for these structures indicate differences in charge exposure that correspond with altered structural arrangement, as seen by differences charge asymmetry (Figure 8B, C). Native HA (3QQB) exhibits the most negative charge at a pH of 5.5. This corresponds to the conditions under which HA is triggered to undergo a rearrangement to the low pH configuration 3QQO. In this form, a great increase in the exposure of positively charged moieties is observed. When this structure is re-neutralized (3QQE) more positively charged groups are retained than on the native configuration. These differences are localized to the region of the protein distal to the viral membrane along with the α–helical hinge region. The structures seen during the process of acidification followed by neutralization agree with the EM measurements which indicate a triggered exposure of additional positively charged moieties.

While the structures used in these models are not flexible unlike the recombinant protein, the differences between them indicate changes that occur when pH induced triggering of HA occurs. The difference in surface potential between the native form and the reneutralized form (Figure 8C) reveals that the asymmetry in charge is not maintained over all conditions, but is most prevalent in the environment characteristic of endosomes where HA-driven membrane fusion occurs. These data indicate that the differences in surface chemistry seen with EM measurements are consistent with previously described conformational change occurring as defined by altered crystal structures.

### Electrophoretic mobility of soluble CD4 does not mirror that of T cells

**Figure 9:**
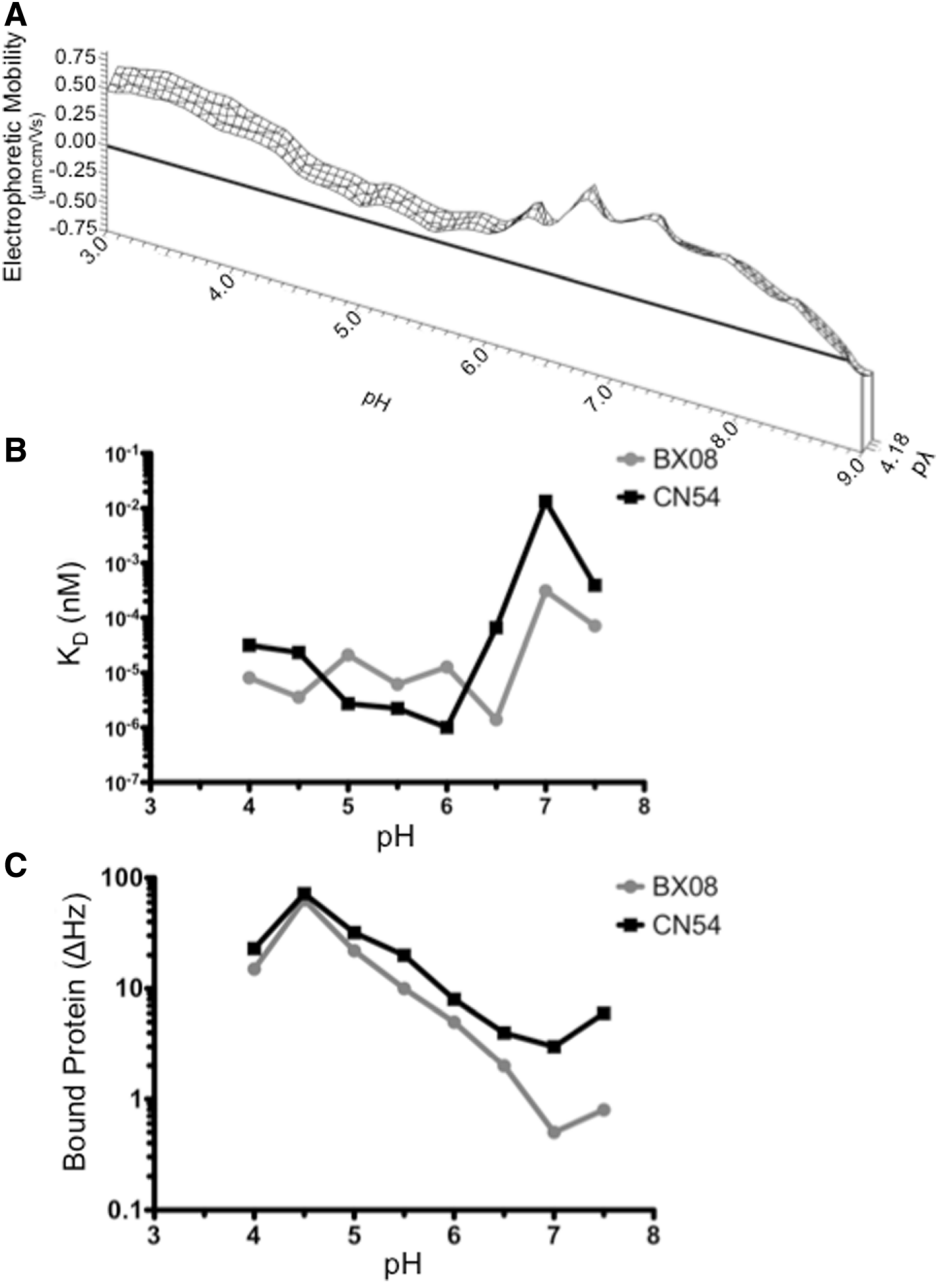
**Electrophoretic mobility and affinity characterization of soluble CD4 (sCD4) across a range of pH.** (**A**) Electrophoretic mobility was measured at 13 pH values ranging from 3.0 to 9.0 for sCD4 in 154 mM NaCl. Data was collected for three titrations, and three measurements were made and averaged at each titration point (n = 27 per titration point). Electrophoretic mobility is positive for all pH conditions examined except pH 9.0 where slightly negative values of −0.15 μmcm/Vs are observed. Surfaces were plotted using direct linear interpolation. (**B**, **C**) Soluble recombinant human CD4 (sCD4) binds to trimeric gp140 in a pH dependent manner. Using an Akubio RapID4 acoustic biosensor, sCD4 is immobilized covalently on a gold coated quartz sensor cassette and trimeric BX08 or CN54 gp140 at concentrations ranging from 1 to 8 μgml^-1^, in a two fold dilution series, are allowed to bind for 3 minutes followed by 5 minutes of dissociation. This binding process was performed across 8 pH values, from 4.0 to 7.5 in 0.5 pH increments. Surfaces were regenerated in between injections by washing with low pH glycine buffer with Tween-20. Affinity constants were calculated according to the Langmuir binding model. Protein bound was measured at the highest gp140 concentration as the change in vibration frequency of the sensor surface. Data shown are the mean of triplicate measurements.

To initiate infection, the Env complex of HIV-1 must sequentially interact with the CD4 molecule on a host cell, followed by a chemokine receptor (CCR5 or CXCR4) before viral fusion and entry. In order to isolate the influence of the primary receptor for the HIV-1 virion from the total surface charge of a cell where many proteins will influence the net behavior, measurements were made on recombinant soluble CD4 (sCD4, Figure 9A). A single value of physiological salinity (pλ 4.1-4.2, 154 mM NaCl) was examined due to scarcity of material. This titration shared some characteristics with that of gp140 trimers. The EM began with a positive value of 0.5 μmcm/Vs at pH 3.0 that decreased up to a pH of 5.0. At this point, the rate of change in EM decreased to near zero, remaining slightly positive at 0.14 μmcm/Vs. Between pH 5.5 and 7.2, the EM increased slightly with the addition of additional anions, reaching a maximum EM of 0.90 μmcm/Vs at pH 6.8; this was followed by a decreasing EM to a pH of 9.0 where slightly negative EM was observed.

Positive EM across a wide range of increasing anion concentration was contrary to our previous observations made using HIV-1 permissive CD4^+^ T cell lines [30,31] but not for the single molecules in this study. CD4^+^ T cell lines had negative EM under most pH and pλ conditions. The CD4 molecule has a positive charge under nearly all conditions examined, outside of the most alkaline environment. The CD4 molecule makes up only a small proportion of total cell surface protein, and its mobility is therefore not the principle determinant of the entire cell surface.

### HIV-1 Env gp140 binding to sCD4 changes throughout the range of pH present at the genital mucosa

To correlate the changes seen in the surface chemistry of the Env protein and the CD4 molecule with functional interaction, kinetic binding studies were performed over a range of pH conditions using an acoustic biosensor. In order to address the physiological relevance of the EM changes seen in the gp140 trimers, their binding to sCD4 was assessed as a function of pH (Figure 9B,C). CN54 and BX08 gp140 glycoproteins were immobilized on sensor cassettes, and soluble CD4 allowed to bind at a range of pH values. Four concentrations of sCD4 were applied to the Env trimers at each pH value, and affinity (K_D_) was calculated according to the Langmuir model of binding. The K_D_ was seen to follow the path of the electrophoretic fingerprint (Figure 9B). A stronger affinity was measured under the conditions where the gp140 was the most negative, in the range from pH 4.0 to 6.0. When the electrophoretic fingerprint showed a large increase in mobility, near neutral pH, there was a substantial loss in binding affinity. The Env from each of the two clades behaved similarly, having the weakest binding at neutral pH. The BX08 gp140 showed its strongest binding to sCD4 at a pH which is consistent with that of cervico-vaginal secretions.

The sCD4 protein bound to Env trimers after the association phase was also quantified for the highest concentration used, 8μgml^-1^ (Figure 9C). The amount of protein bound was also seen to be dependent on the pH of the binding buffer. At the most acidic pH, there was more than ten-fold higher capture of sCD4 than under conditions reflective of serum. Together, these data indicate that the nature of the envelope-receptor interaction was strongly influenced by the environmental pH. However, binding measurements with whole virus interacting with immobilized CD4 at a range of pH conditions did not result in substantive observable binding occurring outside of physiological pH (Data not shown).

### Dynamic electrophoretic fingerprint of purified whole virions

In order to develop a generalized model for the electrochemical relationship between target cells and the infectious agent, viral particles were characterized for electrophoretic mobility. Purified viral stocks of HIV-1 stains BX08 (R5), BaL (R5), and IIIB (X4) were generated, inactivated with aldothiol-2, and depleted of microvesicle contamination.

**Figure 10:**
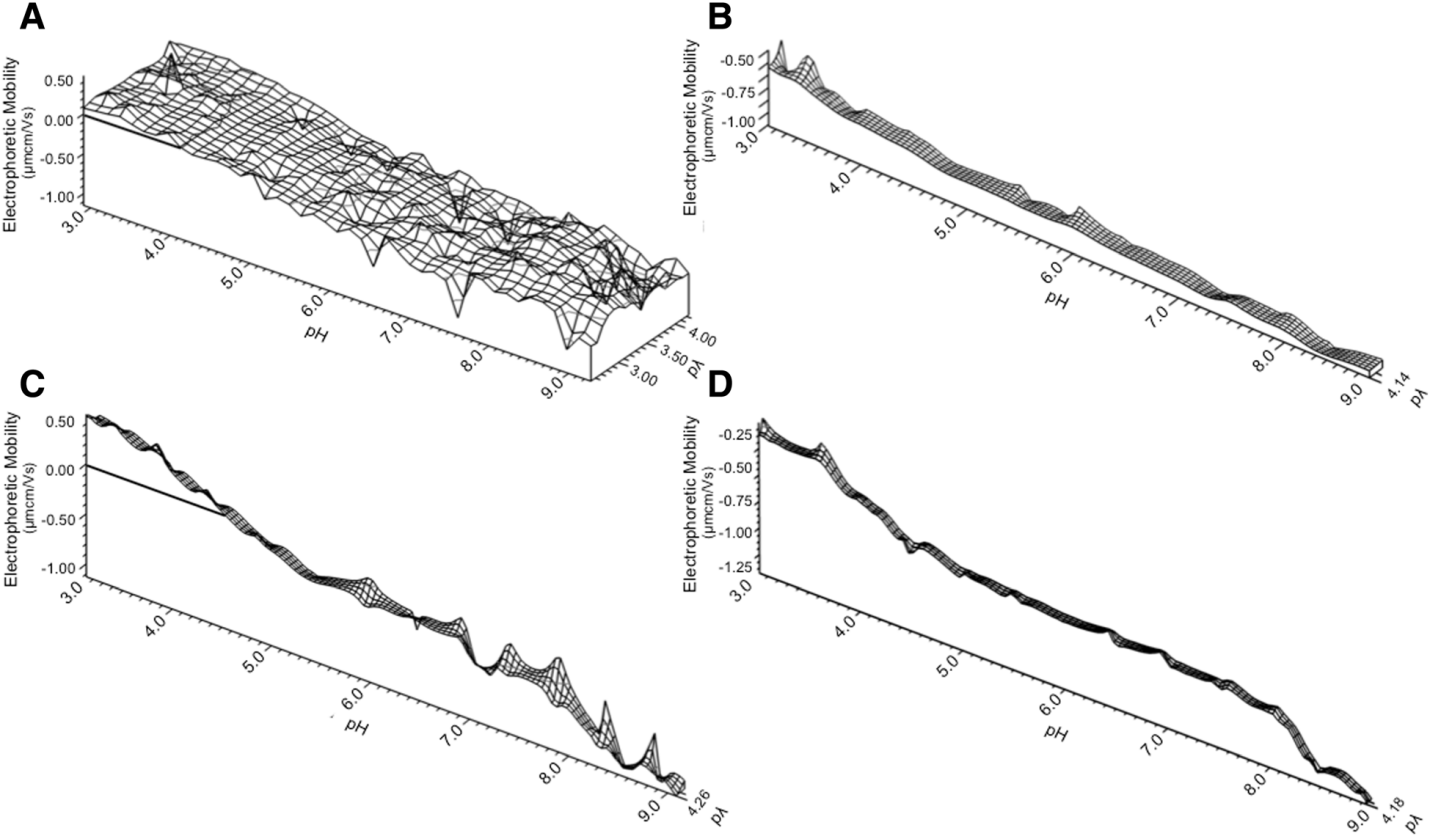
**Partial dynamic electrophoretic fingerprints (DEF) of three purified HIV-1 strains and microvesicles.** (**A**) R5-tropic BX08, (**B**) R5-tropic BaL, and (**C**) X4-tropic IIIB were concentrated, purified and suspended in 154 mM NaCl. (**D**) Microvesicles from activated T cell line were concentrated and suspended in 154 mM NaCl. DEFs were generated from measurements of electrophoretic mobility made at 13 pH values ranging from 3.0 to 9.0 for particles in 154 mM NaCl for each particle type (pλ 4.1-4.2). BX08 virions were examined at 8 NaCl concentrations ranging from 1 to 200 mM NaCl, corresponding to pλ 2.60-4.31. Data shown is the accumulated result of three independent preparations each with independent three titrations performed per batch of virus and once for microvesicles. Three measurements were made at each titration point which were subsequently averaged (n = 27 for virions, 9 for microvesicles at each pH/pλ combination). Data were plotted using direct linear interpolation.

To enable comparisons with the gp120 and gp140 measurements, a complete DEF of BX08 isolated from clinical samples was generated over the same range of conditions as examined with the isolated Env proteins. Whole BX08 virions exhibited a linear decrease in EM as a function of pH (Figure 10A), and was negative under a wide range of conditions. A single IEP was found which did not change substantially from 3.9 at the lowest pλ conditions to 4.3 at the highest pλ. This differs from the behavior of the trimeric Env protein of the same strain which had an island of positivity centered on neutral pH. The magnitude of EM ranged from 0.17 to −0.51 μmcm/Vs at 200 mM NaCl, and expanded to a range of 0.21 to −0.54 μmcm/Vs at 1 mM NaCl. This is a narrower range of EM than that observed with the isolated BX08 gp120 or gp140. A decreased range of EMs indicates that the surface of the whole viral particle is more resistant to protonation and deprotonation effects than the Env molecule alone.

To compare a clinical isolate with a commonly studied laboratory adapted strain, HIV-1 BaL was examined at physiological salinities. At the most acidic pH measured, the BaL virions were still negative in their ζ-potential (Figure 10B), differing from the proteinaceous surfaces studied and indicative of the presence of abundant strongly anionic groups. The BaL virions were uniformly negative and had a strong resistance to their surface being protonated. The ζ-potential did not change any more than that seen with the BX08 strain, ranging from −0.39 to −1.19 μmcm/Vs, which reflects a similar change in the degree of ionization in both strains.

Experiments were also performed with X4 tropic HIV IIIB (Figure 10C), reflective of late stage and cytopathic infection, to see if there was a difference in EM as a function of co-receptor usage at physiological salinity. The X4-tropic virus was more easily protonated than either of the R5 tropic viruses. At the acidic pH range, viral particles exhibit a positive EM, measuring 0.47 μmcm/Vs at pH 3.0. The EM of IIIB virions decreased steadily over the titration and a single IEP was observed at pH 4.35, with a maximum negative EM at pH 9.0 of −1.19 μmcm/Vs. Although the IIIB virions were also negatively charged under most conditions, they were less negative than BaL only reaching the negative charge of BaL virions under the most alkaline conditions examined. However, whether this is a general attribute of viral tropism cannot be conclusively inferred. The ζ-potential of IIIB virions was distinct from that of BX08 and BaL. No change in slope of the EF was seen over the pH titration although there was some localized variability above pH 6.9. Although IIIB and BaL are both laboratory adapted strains and accordingly have different envelope structures than primary clinical isolates, the difference between them were greater than those between the two R5-tropic strains (BaL and BX08).

The shape of the intact virions DEF did not reflect the changes in sign of ζ-potential seen in the isolated gp140 Env measurements. The measurements with BX08 gp120 monomers also were more negative under all conditions compared to the whole purified viral measurements: EM ranged from −0.16 μmcm/Vs to −1.20 μmcm/Vs at a pλ of 4.18.

To compare HIV-1 virions with similar small membrane bodies secreted by activated or apoptotic cells particularly during acute HIV-1 infection, microvesicles were concentrated and characterized in the same manner as for the virus. Microvesicles were produced by activating the uninfected PM-1 T cell line with PHA and IL-2 overnight, collecting the supernatant containing microvesicles and concentrating over a sucrose cushion (omitting the CD45 depletion). Examining the EM of the microvesicles under the same conditions as virions showed that their surface chemistries were quite similar (Figure 10D). Microvesicles had negative EM under all conditions examined, closely resembling BX08 and BaL virions. Overall, there was a stepwise decrease in EM as a function of pH, with a leveling off near −0.80 μmcm/Vs between pH 4.9 and 7.5. This was most similar to the profile of the behavior seen with viral strain BX08 (stable EM between 5.0 and 7.4). Microvesicles produced from highly activated cells share many of the same non-virally encoded host proteins as HIV, the similarity between the two systems implies that the presence of Env on particle surfaces has restricted impact on change in total surface potential.

### Comparing activated and resting primary CD4^+^ T cells reveals changed surface chemistry

**Figure 11:**
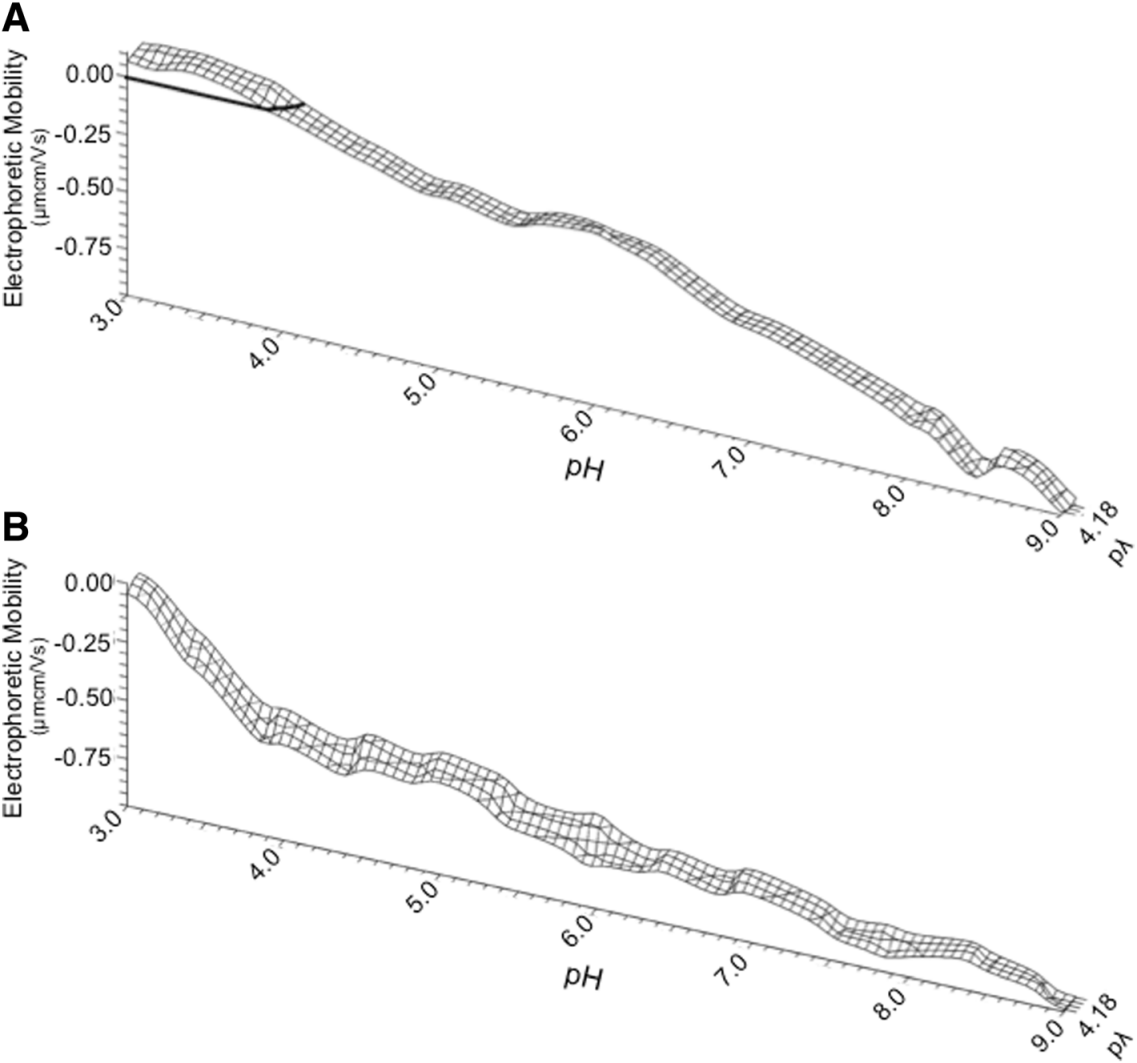
**Partial dynamic electrophoretic fingerprint (DEF) of primary CD4**^**+ **^**T cells.** 10^7^ purified CD4^+^ T cells (**A**) resting or (**B**) activated were suspended in 154 mM NaCl and titrated from pH 3.0 to 9.0 in increments of 0.5 pH units where electrophoretic mobility was measured. Activation was induced by treatment with phytohemagglutinin and interleukin-2 overnight. DEFs were generated from three titrations were made for each of three donors, with three measurements made at each titration point, which were averaged (n = 27 per titration point) and plotted using direct linear interpolation.

In previous studies [30-32], we performed a comprehensive analysis of the surface chemistry of T cell lines commonly used to propagate HIV *in vitro*. Here, we evaluated the surface chemistry of primary CD4^+^ T cells. Resting-state CD4^+^ T cells showed a slightly positive EM at pH 3.0, which decreased steadily with increasing pH (Figure 11A). This reflects the classically described behavior of surface charge changes in a colloidal lipid-based system, but was distinct to that previously reported for CD4^+^ T cell lines [30]. The average standard deviation between each of the three donors was 0.058 μmcm/Vs (range 0.01-0.12). These results demonstrate that the EM of CD4^+^ T cells was largely invariant from donor to donor as well as between titrations. The slope of the DEF as a function of pH was consistent throughout the titration.

A subsequent analysis was performed to determine the effect of cellular activation on the EM of primary CD4^+^ T cells. Purified CD4^+^ T cells were treated with PHA and IL-2 overnight to induce an activated cellular phenotype. The EM of the activated cells, from the same donors, was measured as for resting cells and the resulting partial dynamic fingerprint is shown in Figure 11B. The initial slope was measured as −0.63 μmcm/Vs per pH unit from pH 3.0 to 4.0, and subsequently decreased to −0.12 μmcm/Vs per pH unit when measured over pH 4.0 to 9.0. The EM was more negative under a wider range of conditions for activated cells than resting ones. Interestingly, the slopes of activated CD4^+^ T cells reflect those previously reported for replicating CD4^+^ T cell lines and in particularly the H9 cell line [30,31].

## Discussion

In this study we investigated the cellular and viral components present during infection in conditions that mirror sexual transmission of HIV-1. The use of recombinant Env constructs allowed study of their electrophoretic profile in isolation from non-viral proteins incorporated within the viral membrane. The dynamic electrophoretic fingerprint (DEF) of the three strains of gp120 examined concords with classically described colloidal surfaces [47], exhibiting a steady-state decrease in electrophoretic mobility (EM) over the pH sweep. The lack of any substantial changes in curvature indicates a conformation allowing successive protonation of a fixed array of solvent exposed groups. This observation facilitated comparison to ESP models of defined crystal structures of gp120, which were also seen to undergo a twisted ribbon type titration. The relative simplicity of the monomeric gp120 DEF underscores previous structural and immunogenicity studies indicating that it is incompletely representative of the quaternary structure presented by the functional Env spike expressed on infectious virions [48,49]. Indeed, induced antibody responses to gp120 constructs have low or no affinity for complete Env spikes [50,51] with highly restricted neutralizing activity.

The pliability of the Env spike is likely altered by being expressed as a monomeric protein in solution. According to a recent study [21], when the V1V2 domain and gp41 contacts are removed, the native gp120 core prefers a conformation that is similar to a CD4 bound conformation. However, another recent SAXS study demonstrated that when missing regions such as V1V2 is introduced onto a single monomer gp120 core, the core adopts a different conformation [52]. Some aspects of these conformational changes are considered here by examining a series of gp120 monomer structures by the modeling of ESP. The differences in ESP measurements with and without inclusion of variable loops and glycosylation indicate that these factors are important for determining the degree of protein EM, but are not the principle determinants of surface charge characteristics.

In contrast, trimeric gp140 constructs displaying proper quaternary structure are able to undergo similar conformational changes as functional Env spikes and are therefore considered a significant improvement on monomeric gp120 [53,54]. Indeed, immunization with oligomeric Env induces greater antibody neutralization breadth and potency, where neutralization has been shown to positively correlate with antibody affinity for the trimeric envelope in its native state [55]. However, recombinant trimeric gp140 constructs are still imperfect mimics of functional Env spikes as most lack functional cleavage sequences, while the absence of presentation within a viral membrane excludes any affect of the lipid bilayer on the conformational constraints of Env. Nevertheless, the DEF of trimeric gp140 was very different to that of monomeric gp120 derived from the same viral strain (BX08). Within trimeric Env, the variable loops will be more constrained due to packing and interactions of the monomers and thus difference between strains will have decreased influence on EM relative to the monomer.

The DEF of BX08 gp140 displayed a biphasic pattern. At low pH, the DEF for BX08 gp140 followed a similar trend seen to that of monomeric gp120 with an IEP at pH 4.9 in the low salinity environment which decreased with increasing pλ. The portion of the DEF up to pH 5.0 is typical of other proteinaceous particles characterized by decreasing mobility with the addition of cationic hydroxyl groups and increased amplitude of the DEF at lower salinity environments [33,47]. However, above pHx the behavior of the glycoprotein dramatically changed adopting a strongly positive charge while the solution pH increased. This inflection likely reflects pH-driven conformational changes leading to exposure of previously occluded positively charged moieties within the glycoprotein. Concerted structural rearrangements are not necessary to explain the changes in EM, instead there may be a shift in the balance of energetically preferable sampled states. Conformational heterogeneity of native Env trimers has been proposed by cryotomography studies whereby multiple Env conformations exist in an equilibrium [56].

The DEF of the trimer implies that Env present in the genital mucosa will exist in different conformations or energetic states than that in the blood or semen. The pH of cervico-vaginal mucosal secretions is at the lower end of the scale examined (pH 4.5) where HIV-1 has no substantial decrease in infectivity [57,58]. Alternatively, clathrin mediated endocytosis pathways can also provide acidic environments and a source of cellular ingress for HIV-1 [34,36,59]. Acidification of these environments would induce the same phenotypic changes in Env that we see in the DEF. Under these conditions, gp140 glycoproteins display their most negative characteristics, while the most positive mobility is observed at the pH of the blood (pHy). If conformation or epitope exposure of HIV-1 Env is dependent on pH, as it is for influenza HA, then these compartments would express different phenotypes of Env. In the context of sexual transmission, when slightly alkaline semen raises the pH of acidic cervico-vaginal secretions, the pH within the vagina returns to its pre-coital characteristics quite rapidly [60,61]. Further, while the buffer capacity of semen is greater than that of cervico-vaginal fluid, the pH of the mixture can still confer a decreased EM on Env as virions transit from a seminal carrier to one acidified by lactobacilli.

The quaternary structure of Env trimers has primarily been inferred from analyses of monomers and remains controversial [8,21] although it is accepted that a number of folding events occur during a successful infection event. During the entry process trimeric Env undergoes a large structural transition from a closed, unliganded state to an open state when in complex with CD4. This change is thought to reveal the HR1 domain of gp41 and may also expose epitopes susceptible to neutralization, such as the CD4-induced (CD4i) domain, which are occluded on closed structures. Receptor binding also initiates V1/V2 loop repositioning towards the outside of the trimer and significant reorientations of the V3 loop [62]. All of these folding events would result in the exposure of amino acid chains to which access had been previously restricted. The neutralization sensitivity of viral strains may be related to the activation barrier of trimeric Env to transition to the open state as well as CD4 independence [63]. It is possible that Env in alternative conformations, such as those inducible by low pH, may have different functional characteristics. The CD4 binding site can be rendered more neutralization-sensitive by specific alterations to the surrounding glycan moieties [64], while binding to CD4 possibly serves the additional purpose of cloaking conserved antigenic sites from exposure [65,66]. The observation of an altered DEF from that of the classical surface could also reflect formation of the metastable unliganded conformation without the ligand present.

The conformational changes that occur during viral binding and membrane fusion may not be well represented by the interaction of CD4 and gp140 alone. The absence of co-receptor, as well as the inability to form the six helix bundle, may mask a more complete understanding of Env glycoprotein biochemistry. However, the initial event necessary to initiate HIV-1 infection remains the binding of Env to CD4. Comparisons of HIV with influenza envelope protein HA show a high degree of structural and mechanistic homology [46,67]. The HA protein undergoes conformational change driven by a pH decrease in the endosome exposing the “spring-loaded” domain in HA2 of hemagglutinin for fusion [68,69]. The α-helical folding domains in HA that are seen to experience increases in ESP are also those implicated in forming the fusion intermediate [70]. The N-terminal 23 residues of HA2, which are quite hydrophobic and highly conserved across all serotypes, are shielded before acidification. In the fusion-active state, the newly exposed fusion peptide is projected from a coiled-coil stem, with this process representing a critical stage in HA driven membrane fusion. The shape of the DEF observed with trimeric HA glycoprotein under identical experimental conditions to trimeric Env reveals matching curvature. As pH conditions approach neutral, and the surface charge of gp140 trimers are seen to change from negative to positive, HA trimers also acquire a positive ζ-potential. Further, because HA is known to undergo structural rearrangement in response to pH change and the surface potential profile is similar to that of HIV-1 Env, this raises intriguing questions with respect to the influence of pH on the process of HIV binding and entry in the mucosa. The observation that genetically disparate clade B and clade C gp140 trimers displayed similar DEF and sCD4 binding patterns suggests these results are likely applicable to trimeric Env glycoproteins in general.

The DEF of the CD4 molecule displayed far less variation. The invariant and near uniformly positive behavior of sCD4 differs from models of the surface chemistry colloidal particles being negatively correlated with pH. The CD4 molecule has moderately positive EM over a wide range of pH values. The difference in surface potential, which implies optimal charge attraction, between CD4 and gp140 is maximized at the same pH that the binding kinetics predict. CD4 preferentially interacted with Env at pH values ranging from 4.0 up to 6.0, with the most total binding and highest affinity occurring at a pH of 4.5, which falls within the range of healthy cervico-vaginal secretions. Relative binding affinity correlated with a more negatively charged Env trimer, while conditions which confer a positive ζ-potential on the Env complexes displayed weaker and less avid binding. The changes in binding interaction at diverse pH values are not attributable to CD4 surface charge, which was minimally affected by pH. Considering that a sustained low pH environment is not conducive to HIV-1 replication, it is likely that the conformational changes and binding events we observe are limited to the Env-CD4 interaction. Subsequent entry and internalization into target cells may be differentially influenced by pH.

DEF measurements made on whole purified virions HIV-1_BX08_ and HIV-1_BaL_, (R5-tropic strains), and HIV-1_IIIB_ (X4-tropic) contrasted with that of trimeric gp140 alone, displaying an increasing negative mobility with increasing pH. The DEF of BX08 and IIIB displayed a uniform steady decrease in EM across the pH sweep. This was very similar to the DEF of microparticles and resting primary CD4 T cells. Subtle differences were seen with BaL that displayed a biphasic slope initially decreasing at a rate of −0.41 μmcm/Vs per pH unit that then became more gradual in slope to −0.05 μmcm/Vs per pH unit. Interestingly, the DEF of HIV-1 BaL was more akin to that of activated primary CD4^+^ T cells that were more negative compared to resting cells even though positively charged CD4 is up-regulated on activated cells. In this respect activated cells reflect our previous observations with T cell lines commonly used to propagate HIV [30,31]. These previous studies demonstrated that cell surface proteins are the major determinants of changes in EM as cells stripped of surface protein do not deviate substantially from zero mobility [32]. However, the relative contribution of surface CD4 to the global surface charge which is a sum of all exposed proteins is likely to be small. The similarities between the DEFs of primary T cells and virions likely reflects that virions derive their membrane from lipid raft domains of primary lymphocytes, where host derived proteins within the viral membrane are abundantly more prevalent than the 7–14 viral envelope proteins expressed on each virion. Thus the charge characteristics of incorporated host proteins [71] would confer the dominant influence on the EM of virions.

As surfaces with similar charges repel, for viral particles to interact with primary target cells, CD4 and or other binding ligands must extend beyond the localized surface charge of the cell. The four extracellular domains of CD4 extend from the cell surface by as much as 11.5 nm [72] with the D1 Env-binding domain the furthest from the cell membrane. Env also extends significantly from the viral membrane, with the trimeric protein used in this study measuring 12.4 nm by dynamic light scattering, which is consistent with measurements of Env made on virions by EM tomography [5,25]. For a membranous surface in physiological saline, the thickness of the ionic layer, k (the Debye length - K^-1^) is minimized and charge separation between adjacent proteins can occur. For this system, at a physiological pλ of 4.1-4.2 the Debye length is 1.41 nm. This is less than the length of a single domain of the CD4 molecule or the Env complex, which would extend well past the double layer of the cell membrane and have its own, localized surface chemistry. In a low salinity environment with pλ of 2.60, ionic double layer would extend 17.43 nm and localized charges would average over a larger volume. Compression of the boundary layer with higher ionic strength means that the surface charge of each surface component will be exposed to the solution conditions. Thus, the net negative EM seen on T cell surfaces does not preclude individual cell surface proteins from localized regions of positivity. The CD4 molecule has positive EM across all pH conditions and would not result in electrostatic attractive interactions with a similar positively charged trimeric gp140 at neutral pH, instead relying on ligand/receptor specificity. When the surface charge of gp140 is most negative, the strongest affinity interaction between the two is observed. Although the solute molecules within the boundary layer are dynamic this interaction is possible because of ionic boundary thickness.

## Conclusions

In summary, the key findings of this work give rise to an electrochemical model of the microenvironment in which HIV initiates the sequence of events leading to infection. These data support a variable structure of HIV-1 envelope trimers which are capable of sampling a series of different conformations dependent upon pH of the localized environment. These changes were mirrored by the binding kinetics for CD4 suggesting the balance of CD4’s exposure is highly dependent on the pH and pλ of the viral environment. Such changes in Env conformation are dependent on the proper quaternary structure as exhibited by gp140, as they were not observed with monomeric gp120. Furthermore the changes in binding characteristics were not caused by changes in the charge characteristics of CD4, which was positive across pH. Extension of the functional envelope spike and CD4 beyond the net negative surface ionic layer of interacting virus and T cells may facilitate localized interaction between gp140 trimers and CD4 triggering subsequent fusion events. These data demonstrate the highly dynamic nature of trimeric gp140 as influenced by pH and may have important implications to further our understanding of the molecular interactions involved in HIV-receptor binding at mucosal surfaces. The observation that the conformational state of Env in mucosal environments is likely different from that in other compartments should be taken into consideration during design of vaccination strategies that target virus at the mucosal portals of entry.

## Methods

### Cell culture

PBMCs were isolated from single donor buffy coats by Ficoll gradient isolation. Primary CD4^+^ T cells were purified from PBMCs by sequential depletion of monocytes using CD14-conjugated microbeads (Miltenyi Biotec), followed by positive selection for CD4. Cells were maintained in RPMI 1640 supplemented with 10% heat-inactivated fetal bovine serum, 2 mM L-glutamine, 100 IUml^-1^ penicillin, and 100 μg ml^-1^ streptomycin (complete RPMI). Cells were cultured at 2.5-5.0 × 10^6^ cells ml^-1^. CD4^+^ T cell purity was greater than 98% as determined by FACS analysis of CD3 and CD4 expression, and absence of CD8 and CD14. PM-1 CD4^+^ T cell line (kindly provided by the NIH AIDS Research and Reference Reagent Program) was maintained in complete RPMI and passaged every 3 days.

Primary CD4^+^ T cell activation was induced by the addition of phytohaemagglutinin-L (PHA; Sigma) to cell culture at a final concentration of 5 μg ml^-1^ and IL-2 (100 IU ml^-1^ R&D Systems) overnight or for 24 hours to achieve full activation. Resting cells were left untreated for 24 hours before measurement. Microvesicles were collected by activating PM-1 cells in serum free media overnight in the same way as primary T cells and collecting the supernatant.

### Viral culture and purification

Chronically infected PM-1 cells were established following infection with HIV-1_BaL_ or HIV-1_IIIB_ as previously described [73]. Strain HIV-1_BX08_ was grown in a similar fashion using a Jurkat-tat cell line. Viral production was quantified by viral capsid (p24 antigen) release, as measured by ELISA (Frederick, MD) according to the manufacturer’s protocol. Supernatants were harvested to produce a viral stock when p24 levels were greater than 250 ng ml^-1^. Virions were inactivated by incubation with aldrithol-2 (AT-2) for 1 h at 37°C as previously described [74].

To purify virions and microvesicles from other lipid vesicles produced by activated T cell lines, a concentration and immuno-depletion protocol similar to those previously described was used [75]. Briefly, cell supernatant stocks were layered on top of a 17-25% sucrose solution prepared in phosphate buffered saline (PBS) and spun at 100,000 g in a SW55Ti rotor until pelleted. Supernatants were aspirated completely, and the pellets were resuspended in PBS supplemented with 1% bovine serum albumin (BSA) and 5 mM EDTA (Sigma-Aldrich). Microvesicle preparations were resuspended in 0.2 μm filtered 154 mM NaCl for analysis. To viral preparations, CD45 conjugated magnetic beads (Miltenyi Biotec) were added at a concentration of 10 μl ml^-1^ relative to initial viral stock. This preparation of pelleted lipidic vesicles was incubated at 4°C with gentile mixing for a 4 to 6 hours before depletion of CD45 containing microvesicles on magnetic columns according to the manufacturer’s instructions. Immune depleted virions were pelleted at 250,000 × g on 25% sucrose cushions for 30–60 minutes and resuspended in 0.2 μm filtered 154 mM NaCl for analysis.

Viral purity was confirmed by the presence of a single and uniform size distribution as measured by dynamic light scattering and by western blot to confirm the absence of CD45. Preparations had a Z-average diameter of 120.2 nM with a polydispersity index of 0.150, in agreement with previous descriptions of viral preparations [75,76].

### Recombinant proteins

Recombinant HIV-1 Env BX08 gp120 and trimeric BX08 gp140 were produced by Simon Jeffs, Sueli Vieira and Amelia Fuertes of Imperial College London by transfection of CHO cells and affinity purification as previously described [27]. Trimeric CN54 gp140 was provided by Polymun Scientific GmbH (Vienna, Austria). The Env constructs used in this and subsequent experiments were determined to be properly folded by their ability to bind to CD4 and a panel of well-characterized Env specific monoclonal antibodies [27,28]. Additional Env constructs DU123 gp140CF, 1086C gp140C, 63521 gp140, JRFL gp140, A244 gp120Δ11 monomer, and RV144 427200 gp120Δ11 monomer were generated by transfection of 293 T or 293 F cells and purified by size exclusion chromatography and were generously provided by Hua-Xin Liao and Barton F Haynes (Duke Human Vaccine Institute, Duke University). Recombinant soluble human CD4 (sCD4) was kindly provided by the NIH AIDS Research and Reference Reagent Program. Soluble influenza hemagglutinin (HA, H3N2 strain) purified by bromelain cleavage was kindly provided by Prof. John McCauley (MRC UK, Mill Hill).

### Electrophoretic mobility measurements

Electrophoretic mobility (EM, also zeta (ξ)-potential) measurements were made using a Malvern Zetasizer NanoZS operating in the fast field reversal mode (phase analysis light scattering, PALS) equipped with an MPT-2 automatic titrator. The ξ-potential (in units of mV) may be obtained via the Henry equation, by multiplying the reported mobilities by the constant 12.68; the assumption that the Smoluchowski approximation applies is not unreasonable given the size of the systems under examination and the solution ionic strength [77]. This implies that particles sizes are greater than 2 nm in diameter and much smaller than the flow cell, and that the electrolyte concentration molarity is greater than 2 × 10^-3^. All titrations were performed in triplicate, with three measurements made at each 0.5 pH increment. Samples for measuring the electrophoretic mobility of CD4^+^ T cells were prepared by washing cells repeatedly in PBS, and suspending in 154 mM NaCl at a concentration of 1×10^6^ cells ml^-1^. 10 ml of cells were used for each titration, and three titrations were made for each donor. Cells were only used once for electrophoretic mobility measurements and only when they were >95% viable as assessed by trypan blue exclusion. Viral preparations were prepared by diluting purified virions to a concentration of 500 ng ml^-1^ p24-gag, as determined by ELISA.

For all experiments, in order to model the sexual transmission event whereby the pH within the female reproductive tract increases, measurements were made using pH titrations from 3.0 to 9.0 in increments of 0.5 pH units. The measurements were initially made at NaCl concentrations of 1.0, 5.0, 10.0, 50.0, 100.0, 154.0, and 200.0 mM. After examining the distribution of the data, when graphed as pλ, 20.0 mM was added to give more evenly distributed data sets. Data was collected until three titrations were made for each condition, which in some cases resulted in more than three partial titrations being made, due to titrator error. All data were included in analysis, unless the measurement failed to meet the quality criteria value of 0.80, as determined by the ZetaSizer software.

Dynamic electrophoretic fingerprints were plotted using Surfer 6 software (Golden Software; Golden, CO) (Figures 1, 2, 4, 5, 9, 11), or IDL 7.0 (Exelis; Boulder, CO) (Figures 6, 7, 10), using standard gridding algorithms. No smoothing was applied to the measured set of mobilities, so there is a degree of noise in local data which represents the experimental deviation in the measurements.

### Protein binding assay

In order to assess the binding of sCD4 to Env proteins, a Rap-ID 4 acoustic biosensor was used. This platform determines response as the decrease in frequency of a vibrating quartz crystal as material binds, or as an increase in resonant frequency as dissociation occurs over time. BX08 gp120 and 140, and CN54 gp140 were prepared at a concentration of 50 μg ml^-1^ in sodium acetate buffer, optimized at pH 4.5 and covalently bound to a gold coated quartz crystal using an ethyl (dimethylaminopropyl) carbodiimide hydrochloride (EDC) with N-hydroxysulfosuccinimide (NHS) amine coupling. Unreacted ester groups were neutralized with ethanolamine blocking. sCD4 was prepared at 1.0, 2.0 and 4.0 and 8.0 μg ml^-1^ in phosphate-citrate buffer at pH values ranging from 4.0 to 7.5 in increments of 0.5 pH units. Protein was allowed to flow over the immobilized Env protein for 3 minutes at a flow rate of 25 μl min^-1^ and then allowed to dissociate over 5 minutes. The gp140 surface was regenerated by a 3 minute wash with 100 mM glycine at pH 2.5 with 0.05% Tween-20.

### Modeling of surface electrostatic potential

The PDB structure files were prepared at a range of pH values using the PDB2PQR framework [78] with protonation states for all residues determined using PROPKA3.0 [79] program. ESP calculations were performed by numerically solving the full nonlinear form of the Poisson-Boltzmann equation using the APBS software v1.4 at a temperature of 310 K with standard parameters. ESP grid sizes and granularities were determined using the psize.py script supplied with the APBS software. Partial charges and van der Waals parameters were taken from the AMBER 99 forcefield [80] for protein residues, and the GLYCAM_06h forcefield [81] for glycan residues. The solvent accessible surfaces (SAS) of all structures were determined using the “measure” function of VMD v1.9.1 [82] using a 0.14 nm radius. The mean surface potential (MSP) was calculated by linearly interpolating between all immediately neighboring ESP grid values along the SAS, summing across all SAS grid locations, and dividing by the total surface area. An additional measure of the overall surface potential, charge asymmetry (A), was calculated by taking the sum of all positively charged SAS grid values, subtracting the sum of all negatively charged SAS grid values, and then dividing by the sum of the absolute value of all SAS grid values. This value is effectively the fraction of positive surface charges minus the fraction of negative surface charges, hence having a range of [−1,1]. Surface residue and surface ESP images for individual calculations were rendered using the PyMOL Molecular Graphics System, version 1.4.1, Schrodinger, LLC [83]. Three-dimensional ribbon plots of the mean surface potential and charge asymmetry across all pH and pλ values were created using the “lattice” package [84] for R v2.14.1 [85].

### Ethics statement

PBMC used in this study were isolated from anonymous single donor buffy coats, obtained from the NHS Blood and Transplant Service, where blood was collected through normal blood donation from volunteers who have given written informed consent for non clinical use. Use of PBMC in this study was approved by the Research Ethics Committee of Wales (07/MRE09/54).

## Abbreviations

CD4i: CD4-induced; DEF: Dynamic electrophoretic fingerprint; EM: Electrophoretic mobility; Env: Envelope glycoprotein; ESP: Electrostatic potential; HA: Haemagglutinin; IEP: Isoelectroc point; LZM: Line of zero mobility; pHx: pH of female reproductive tract; pHy: pH of blood; sCD4: Soluble CD4; SAS: Solvent accessible surface.

## Competing interests

The authors declare that they have no competing interests.

## Authors’ contributions

DS purified virus, carried out electrophoretic mobility and binding measurements, analyzed data, and prepared the manuscript. PR carried out electrophoretic mobility measurements and revised the manuscript. DK cultured and purified virus stocks and purified cells. GC analyzed data, wrote plotting procedures for electrophoretic mobility data and revised the manuscript. SJ prepared initial protein expression systems, generated and purified Env proteins and revised the manuscript. JLP and SG performed computational molecular modeling and revised the manuscript. RS designed experiments, analyzed data and revised the manuscript. All authors read and approved the final manuscript.

## Supplementary Material

Additional file 1: Figure 1 Solvent accessible surface and mean surface potential plots for 6 different gp120 structures. 2NY7 is the crystal structure of the b12 bound gp120 core, and 2NY7 (G) is the same structure with glycan residues added to the surface. 1RZK is the CD4 bound gp120 core, and 1RZK (L) is the same structure with the addition of modeled variable loop regions. 2BF1 is the unliganded gp120 core from SIV, and 2BF1 (L) is the same structure with the addition of modeled variable loop regions. Click here for file

Additional file 2: Figure 2 Solvent accessible surface and charge asymmetry plots for 6 different gp120 structures. 2NY7 is the crystal structure of the b12 bound gp120 core, and 2NY7 (G) is the same structure with glycan residues added to the surface. 1RZK is the CD4 bound gp120 core, and 1RZK (L) is the same structure with the addition of modeled variable loop regions. 2BF1 is the unliganded gp120 core from SIV, and 2BF1 (L) is the same structure with the addition of modeled variable loop regions. Click here for file

Additional file 3: Figure 3 Mean surface potential and charge asymmetry differences between each of the three gp120 crystal structures studied, 2NY7, 1RZK, and 2BF1, and their corresponding modified versions, 2NY7(G), 1RZK(L), and 2BF1(L). Click here for file

Additional file 4: Figure 4 Mean surface potential and charge asymmetry differences among each of the three gp120 crystal structures studied, 2NY7, 1RZK, and 2BF1. Click here for file

Additional file 5: Figure 5 Electrostatic surface potential differences among each of the 6 gp120 structures. Click here for file

Additional file 6: Figure 6 Remaining charge asymmetry differences among each of the 6 gp120 structures. Click here for file

Additional file 7: Figure 7 Solvent accessible surface models of 6 different gp120 structures colored by electrostatic surface potential across a wide range of pH values. All structures have been aligned to the 2NY7 structure (as shown in Figure 3A) to facilitate visual comparison between the different conformations and identification of structural features responsible for changes in the electrostatic surface potential. Click here for file

Additional file 8: Figure 8 Dynamic electrophoretic fingerprint (DEF) of trimeric BX08 gp140. Electrophoretic mobility was measured at 13 pH values ranging from 3.0 to 9.0 and 8 concentrations of NaCl from 1 mM to 200 mM, which corresponds to pλ of 2.60 to 4.35. (A) Postage stamp plot showing distribution of data collection. Each cross represents the average pH and pλ of three electrophoretic mobility measurements. (B) Contour plot of the DEF generated from the data collected in (A). Line of zero mobility (LZM) indicated by bolding show the isoelectric points. (C) Overlay of postage stamp and DEF shows data distribution. Click here for file

Additional file 9: Figure 9 Dynamic electrophoretic fingerprint (DEF) of trimeric CN54 gp140. Electrophoretic mobility was measured at 13 pH values ranging from 3.0 to 9.0 and 8 concentrations of NaCl ranging from 1 mM to 200 mM, which corresponds to a pλ of 2.60 to 4.35. (A) Postage stamp plot showing distribution of data collection. Each cross represents an average pH and pλ of three electrophoretic mobility measurements. (B) Contour plot of the DEF generated from the data collected in (A). Lines of zero mobility (LZM) indicated by bolding show the isoelectric points. (C) Overlay of postage stamp and DEF shows data distribution. Click here for file

Additional file 10: Figure 10 Mean surface potential (A) and differences in mean surface potential (B) for the three HA trimer crystal structures: 3QQB, 3QQE, and 3QQO. Click here for file

Additional file 11: Figure 11 Solvent accessible surface models of the three HA trimer crystal structures colored by electrostatic surface potential across a wide range of pH values. All structures have been aligned to the 3QQB structure to facilitate visual comparison between the different conformations and identification of structural features responsible for changes in the electrostatic surface potential. Click here for file
