# Supplementary figures and images for: Dynamic electrophoretic fingerprinting of the HIV-1 envelope glycoprotein
Source: Retrovirology. 2013 Mar 20;10:33. doi: 10.1186/1742-4690-10-33 (PMC3648349; doi:10.1186/1742-4690-10-33)

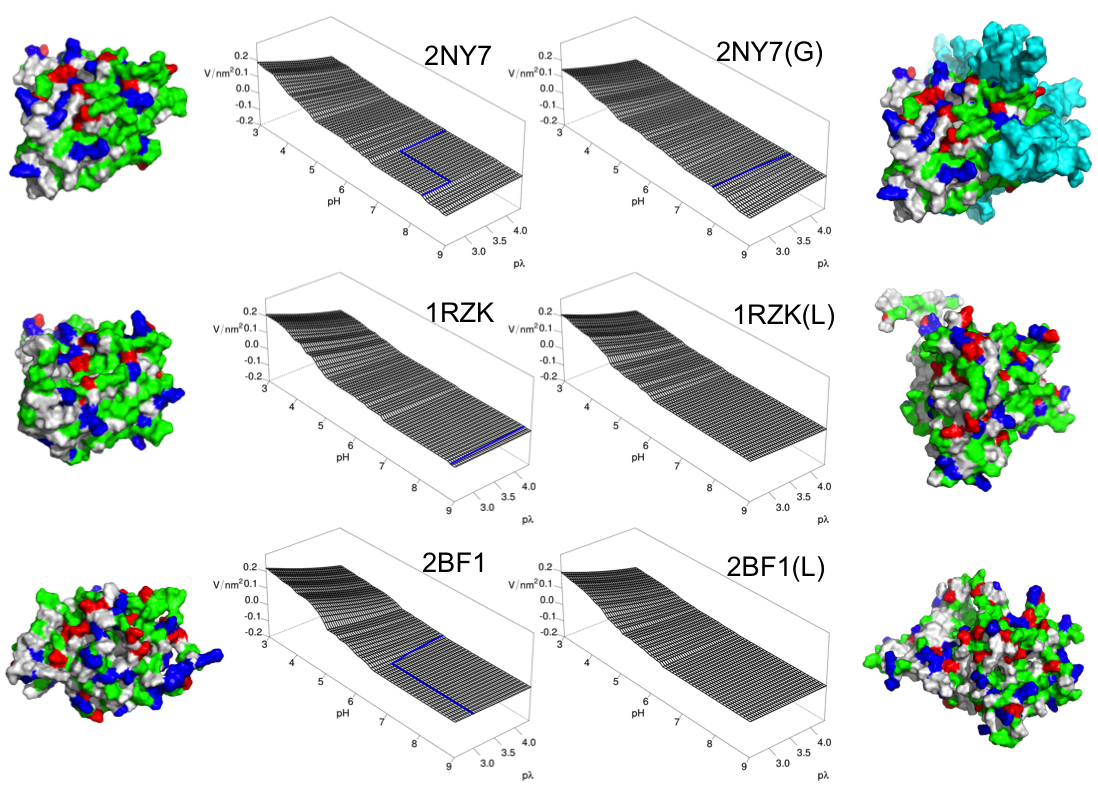

Supplement: Additional file 1: Figure 1 — Solvent accessible surface and mean surface potential plots for 6 different gp120 structures. 2NY7 is the crystal structure of the b12 bound gp120 core, and 2NY7 (G) is the same structure with glycan residues added to the surface. 1RZK is the CD4 bound gp120 core, and 1RZK (L) is the same structure with the addition of modeled variable loop regions. 2BF1 is the unliganded gp120 core from SIV, and 2BF1 (L) is the same structure with the addition of modeled variable loop regions. [file 1742-4690-10-33-S1.tiff]

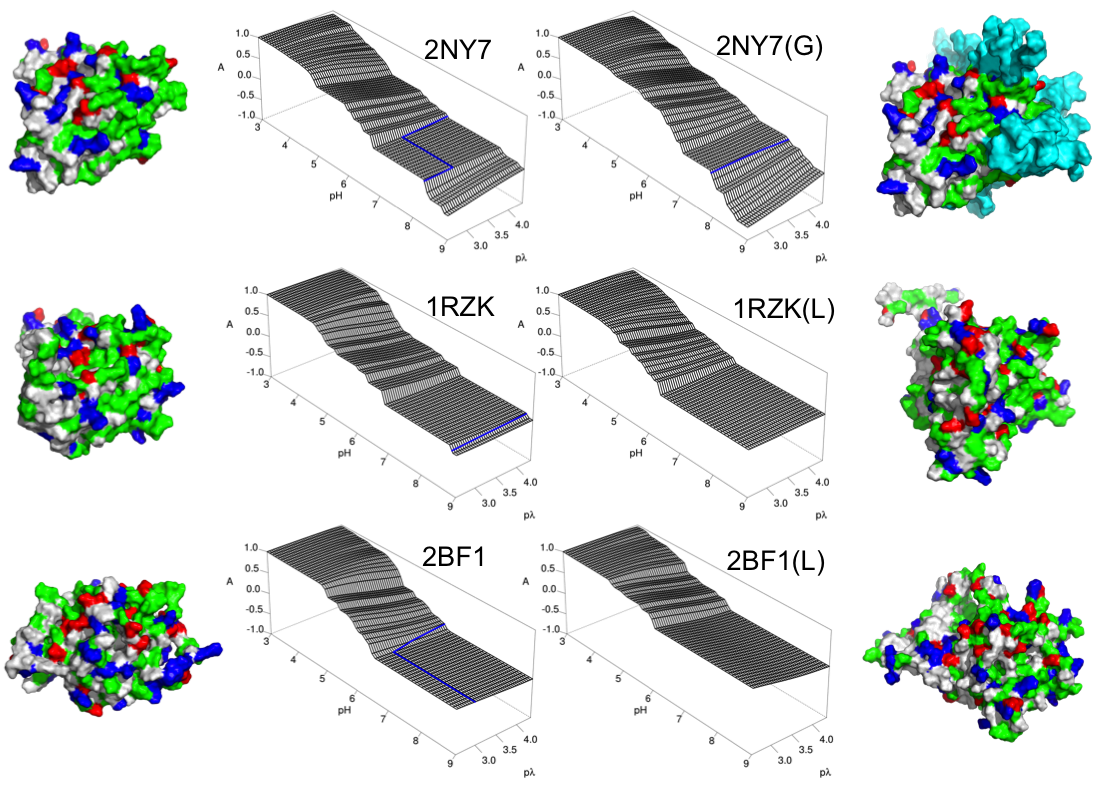

Supplement: Additional file 2: Figure 2 — Solvent accessible surface and charge asymmetry plots for 6 different gp120 structures. 2NY7 is the crystal structure of the b12 bound gp120 core, and 2NY7 (G) is the same structure with glycan residues added to the surface. 1RZK is the CD4 bound gp120 core, and 1RZK (L) is the same structure with the addition of modeled variable loop regions. 2BF1 is the unliganded gp120 core from SIV, and 2BF1 (L) is the same structure with the addition of modeled variable loop regions. [file 1742-4690-10-33-S2.tiff]

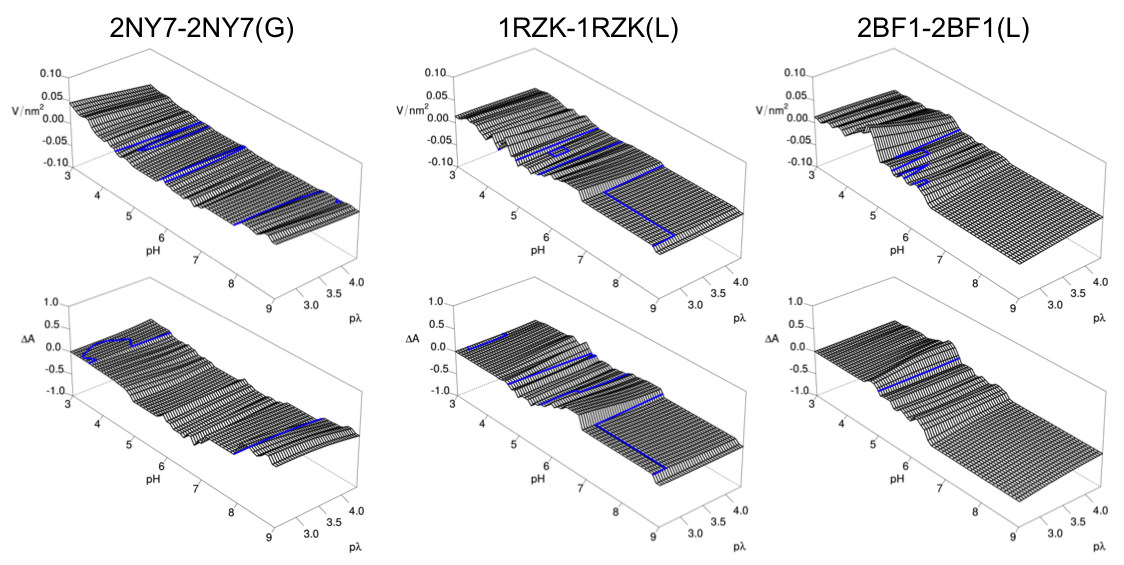

Supplement: Additional file 3: Figure 3 — Mean surface potential and charge asymmetry differences between each of the three gp120 crystal structures studied, 2NY7, 1RZK, and 2BF1, and their corresponding modified versions, 2NY7(G), 1RZK(L), and 2BF1(L). [file 1742-4690-10-33-S3.tiff]

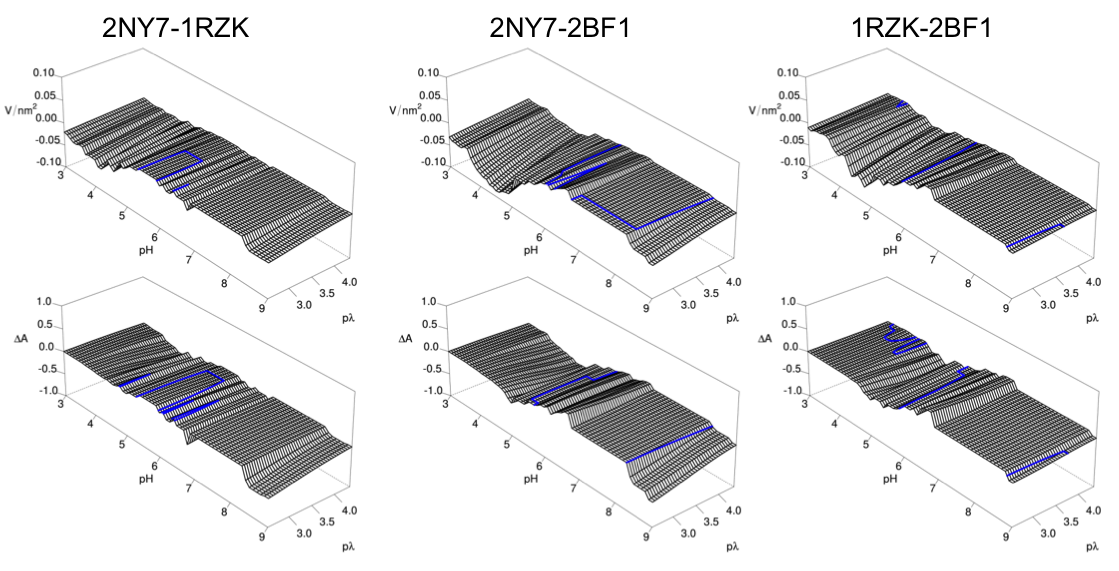

Supplement: Additional file 4: Figure 4 — Mean surface potential and charge asymmetry differences among each of the three gp120 crystal structures studied, 2NY7, 1RZK, and 2BF1. [file 1742-4690-10-33-S4.tiff]

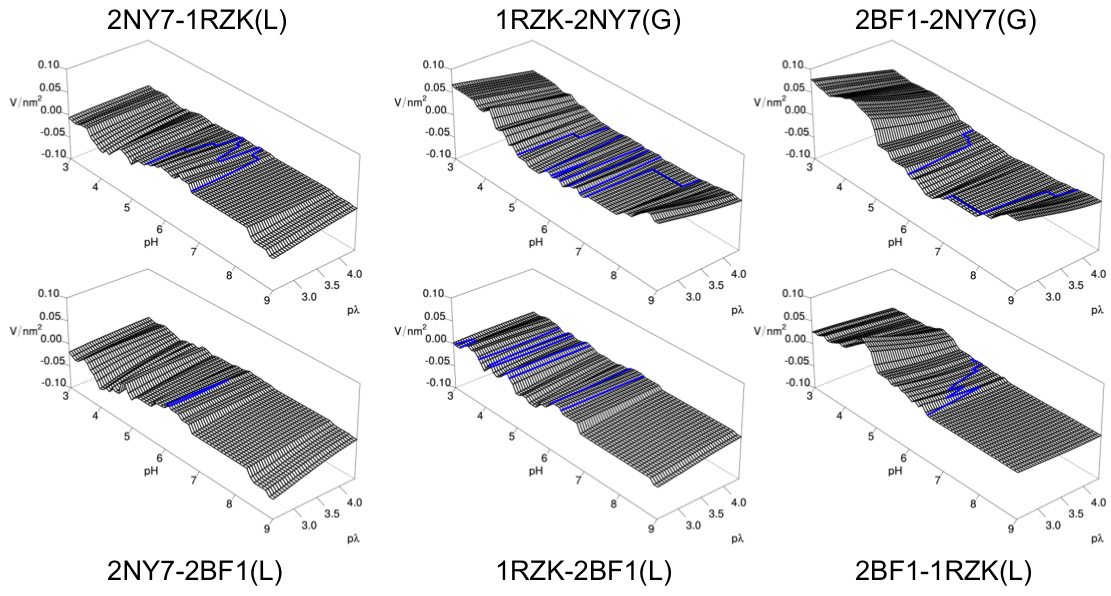

Supplement: Additional file 5: Figure 5 — Electrostatic surface potential differences among each of the 6 gp120 structures. [file 1742-4690-10-33-S5.tiff]

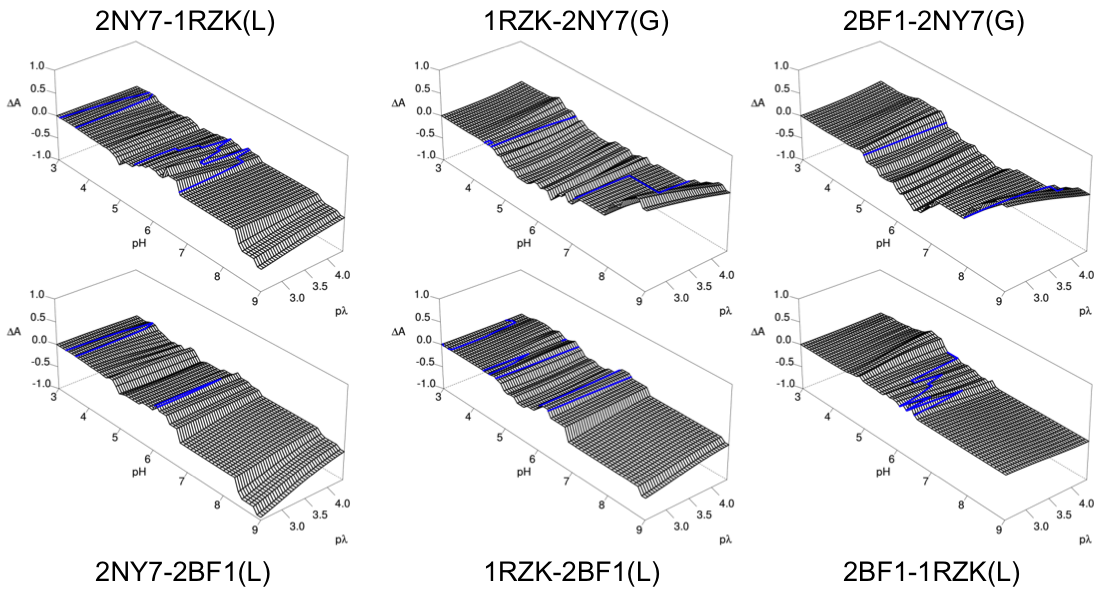

Supplement: Additional file 6: Figure 6 — Remaining charge asymmetry differences among each of the 6 gp120 structures. [file 1742-4690-10-33-S6.tiff]

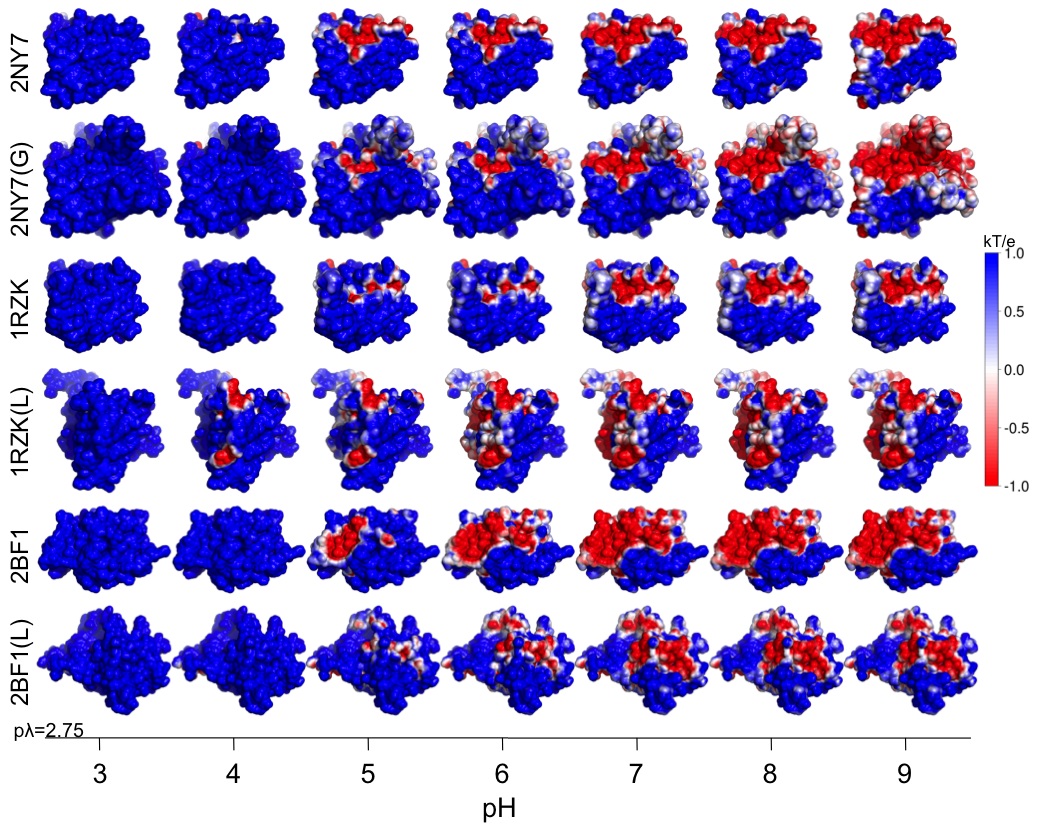

Supplement: Additional file 7: Figure 7 — Solvent accessible surface models of 6 different gp120 structures colored by electrostatic surface potential across a wide range of pH values. All structures have been aligned to the 2NY7 structure (as shown in Figure 3A) to facilitate visual comparison between the different conformations and identification of structural features responsible for changes in the electrostatic surface potential. [file 1742-4690-10-33-S7.tiff]

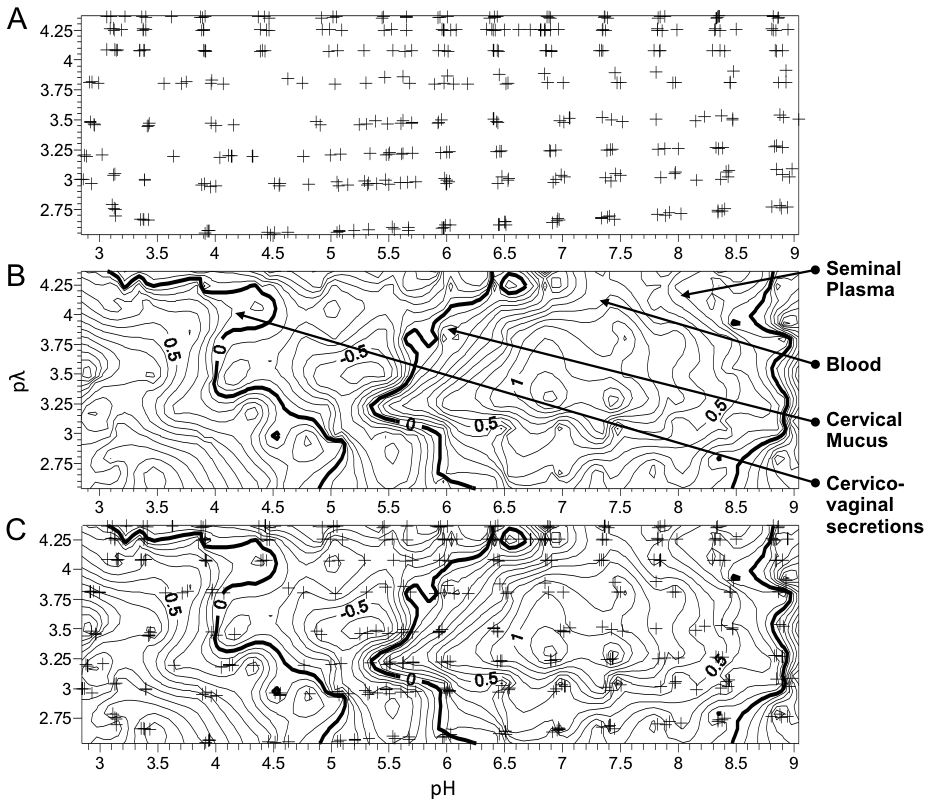

Supplement: Additional file 8: Figure 8 — Dynamic electrophoretic fingerprint (DEF) of trimeric BX08 gp140. Electrophoretic mobility was measured at 13 pH values ranging from 3.0 to 9.0 and 8 concentrations of NaCl from 1 mM to 200 mM, which corresponds to pλ of 2.60 to 4.35. (A) Postage stamp plot showing distribution of data collection. Each cross represents the average pH and pλ of three electrophoretic mobility measurements. (B) Contour plot of the DEF generated from the data collected in (A). Line of zero mobility (LZM) indicated by bolding show the isoelectric points. (C) Overlay of postage stamp and DEF shows data distribution. [file 1742-4690-10-33-S8.tiff]

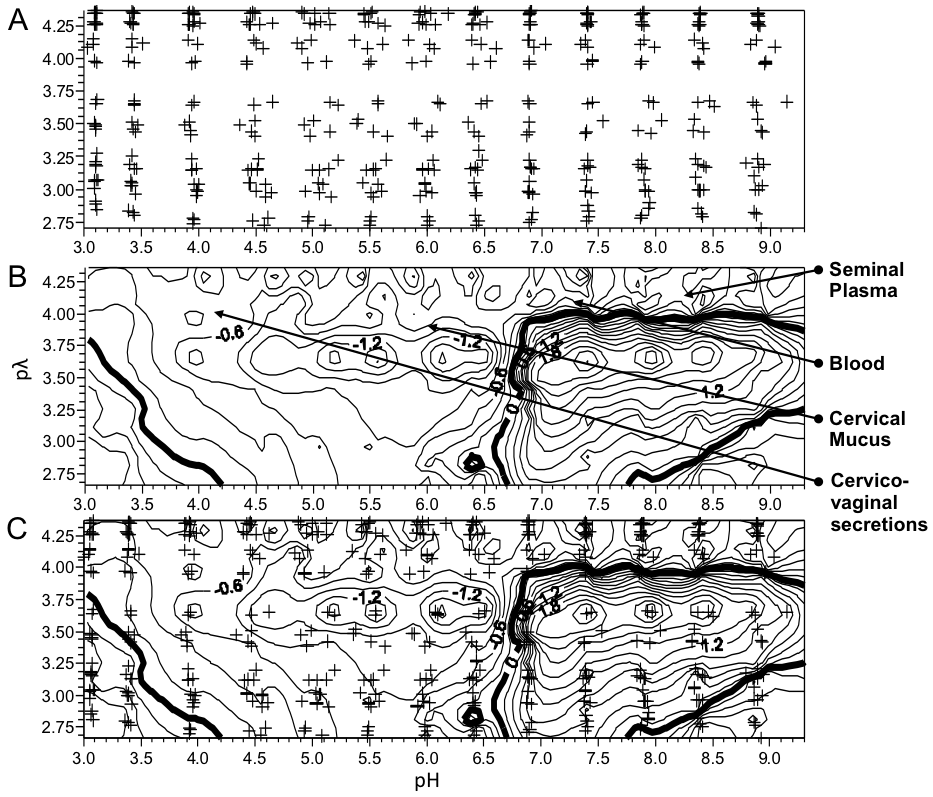

Supplement: Additional file 9: Figure 9 — Dynamic electrophoretic fingerprint (DEF) of trimeric CN54 gp140. Electrophoretic mobility was measured at 13 pH values ranging from 3.0 to 9.0 and 8 concentrations of NaCl ranging from 1 mM to 200 mM, which corresponds to a pλ of 2.60 to 4.35. (A) Postage stamp plot showing distribution of data collection. Each cross represents an average pH and pλ of three electrophoretic mobility measurements. (B) Contour plot of the DEF generated from the data collected in (A). Lines of zero mobility (LZM) indicated by bolding show the isoelectric points. (C) Overlay of postage stamp and DEF shows data distribution. [file 1742-4690-10-33-S9.tiff]

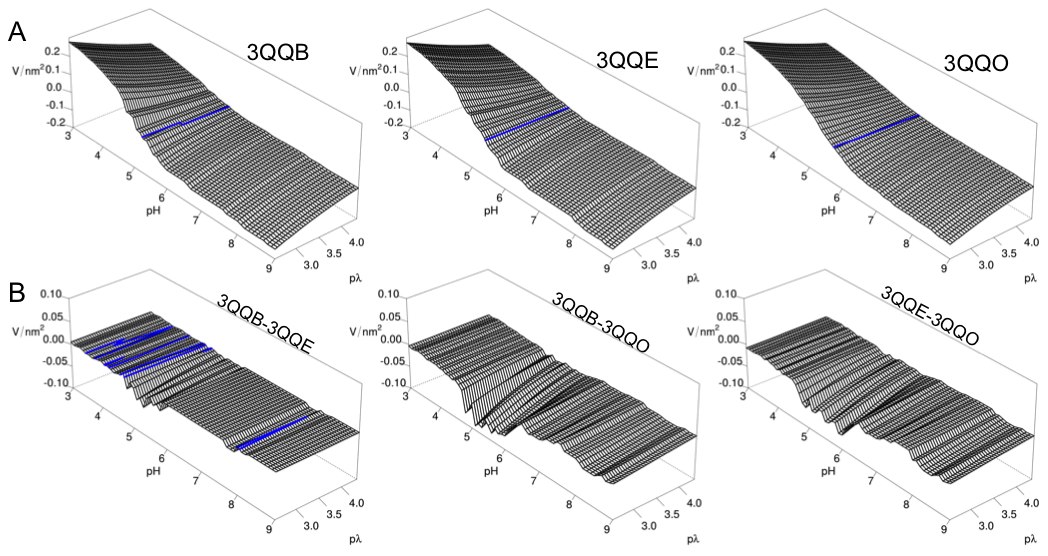

Supplement: Additional file 10: Figure 10 — Mean surface potential (A) and differences in mean surface potential (B) for the three HA trimer crystal structures: 3QQB, 3QQE, and 3QQO. [file 1742-4690-10-33-S10.tiff]

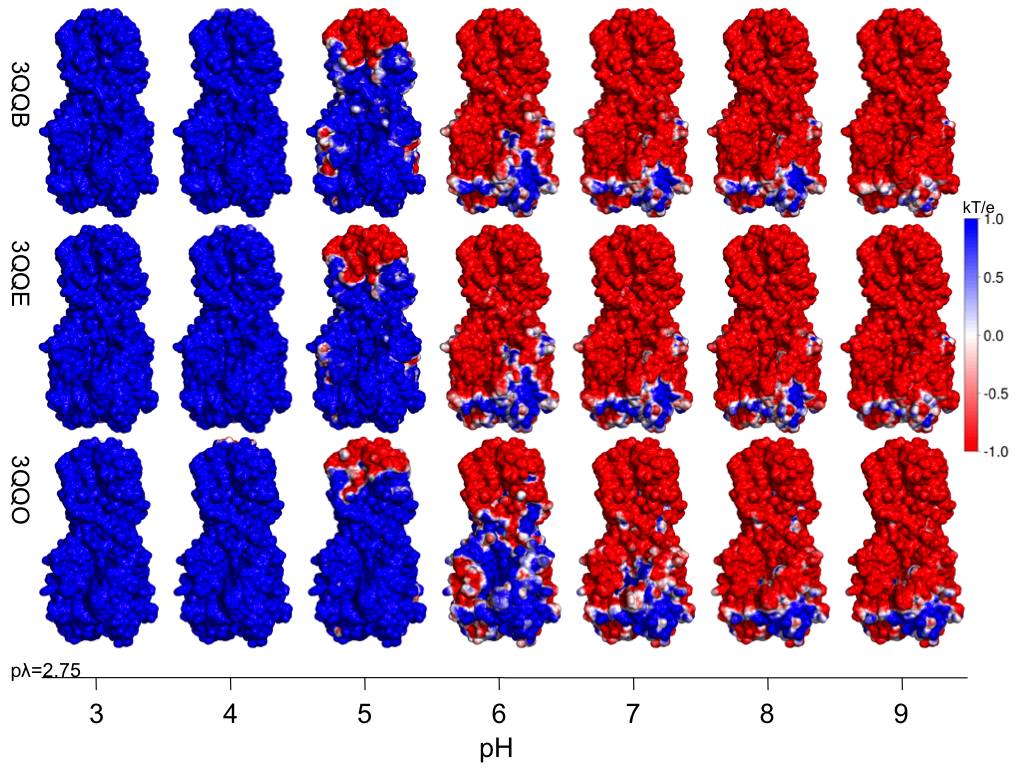

Supplement: Additional file 11: Figure 11 — Solvent accessible surface models of the three HA trimer crystal structures colored by electrostatic surface potential across a wide range of pH values. All structures have been aligned to the 3QQB structure to facilitate visual comparison between the different conformations and identification of structural features responsible for changes in the electrostatic surface potential. [file 1742-4690-10-33-S11.tiff]
